# Alcohol-induced damage to the fimbria/fornix reduces hippocampal-prefrontal cortex connection during early abstinence

**DOI:** 10.1186/s40478-023-01597-8

**Published:** 2023-06-21

**Authors:** Laura Pérez-Cervera, Silvia De Santis, Encarni Marcos, Zahra Ghorbanzad-Ghaziany, Alejandro Trouvé-Carpena, Mohamed Kotb Selim, Úrsula Pérez-Ramírez, Simone Pfarr, Patrick Bach, Patrick Halli, Falk Kiefer, David Moratal, Peter Kirsch, Wolfgang H. Sommer, Santiago Canals

**Affiliations:** 1 Instituto de Neurociencias, Consejo Superior de Investigaciones Científicas and Universidad Miguel Hernández, Sant Joan d’Alacant, Alicante, Spain; 2 Center for Biomaterials and Tissue Engineering, Universitat Politècnica de València, Valencia, Spain; 3 Institute of Psychopharmacology, Central Institute of Mental Health, Medical faculty Mannheim, University of Heidelberg, Mannheim, Germany; 4 Department of Addiction Medicine, Department of Clinical Psychology, Medical Faculty Mannheim, Central Institute of Mental Health, University of Heidelberg, Mannheim, Germany; 5 Department of Psychology, University of Heidelberg, Heidelberg, Germany; 6 Present Address: Radiation Science and Biomedical Imaging, University of Sherbrooke, Sherbrooke, Québec Canada

**Keywords:** Fimbria, Fornix, DTI, Diffusion, White matter, AUD, Alcohol, Prefrontal cortex, Hippocampus, Memory, Flexibility, Cognitive test, TMT

## Abstract

**Introduction:**

Alcohol dependence is characterized by a gradual reduction in cognitive control and inflexibility to contingency changes. The neuroadaptations underlying this aberrant behavior are poorly understood. Using an animal model of alcohol use disorders (AUD) and complementing diffusion-weighted (dw)-MRI with quantitative immunohistochemistry and electrophysiological recordings, we provide causal evidence that chronic intermittent alcohol exposure affects the microstructural integrity of the fimbria/fornix, decreasing myelin basic protein content, and reducing the effective communication from the hippocampus (HC) to the prefrontal cortex (PFC). Using a simple quantitative neural network model, we show how disturbed HC-PFC communication may impede the extinction of maladaptive memories, decreasing flexibility. Finally, combining dw-MRI and psychometric data in AUD patients, we discovered an association between the magnitude of microstructural alteration in the fimbria/fornix and the reduction in cognitive flexibility. Overall, these findings highlight the vulnerability of the fimbria/fornix microstructure in AUD and its potential contribution to alcohol pathophysiology.

**Summary:**

Fimbria vulnerability to alcohol underlies hippocampal-prefrontal cortex dysfunction and correlates with cognitive impairment.

**Supplementary Information:**

The online version contains supplementary material available at 10.1186/s40478-023-01597-8.

## Introduction

Alcohol drinking is a popular activity in our societies, yet it is responsible for more than 2.5 million deaths world-wide each year. Alcohol dependence is a fundamental feature of alcohol use disorders (AUD) and its main therapeutic target [1]. It is characterized by a gradual reduction in cognitive control over alcohol-related activities. A limited ability of the individuals to learn new environmental contingencies, and the impaired response inhibition to stimuli previously associated to alcohol consumption, stabilizes maladaptive behaviors leading to uncontrolled drinking cycles [2]. Despite its clinical relevance and a strongly growing body of literature, we still have a limited understanding of the neuroadaptations underlying this aberrant behavior, which result in a high relapse vulnerability during alcohol abstinence [3].

A large number of brain alterations has been associated to AUD [4–6], many of which persist during long periods of abstinence [7, 8]. A causal link between alcohol and many of these alterations, however, is difficult to stablish due to the inherent complexity of AUD cohorts, with distinct genetic and psychosocial factors, personality traits and disease trajectories affecting these patients, together with many comorbid factors, including poly-consumption and medication. In this context, animal models with translational value bring us closer to causality and mechanistic understating of alcohol pathophysiology. In a recent study, we have demonstrated a causal link between alcohol drinking and white matter alterations measured by diffusion-weighted (dw) MRI, being this result comparable between chronically drinking alcohol-preferring rats and recently detoxified AUD patients [9]. This finding was consistent with previous studies in AUD [10–12]. Interestingly, though, in our study we were able to demonstrate that these alterations progressed after alcohol withdrawal and during at least six weeks of abstinence in both species [9], suggesting a possible contribution to relapse vulnerability. However, the functional consequences of this progressing white matter alteration are not known.

Although microstructural alterations in the white matter of AUD patients are widespread, they have been more consistently reported in the corpus callosum, the fimbria/fornix, internal and external capsules and cingulate and longitudinal fasciculi [9, 11–14]. Corpus callosum and the fornix were also highlighted in our recent translational study with AUD patients and the genetically selected alcohol-preferring msP rat line [9], and microstructural alterations in the fornix were reported in outbred Wistar rats after binge alcohol drinking [15]. Being the fimbria-fornix the pathway connecting the prefrontal cortex (PFC) and hippocampus (HC) [16], its alcohol-driven microstructural alteration during abstinence may be hypothesized as an important contribution to behavioral inflexibility, common in AUD patients. Indeed, the HC-PFC connectivity plays a well-documented and fundamental role in memory formation and extinction, executive function and emotional processing [17–21].

In this work, to investigate the functional consequences of fimbria/fornix damage in AUD, we used an established rat model consisting of outbred Wistar rats receiving a chronic intermittent exposure (CIE) to alcohol vapor, which leads to intoxication levels similar to those seen in clinical alcohol addiction. This condition, known as post-dependent (PD), represents a ‘relapse-prone’ state of brain networks during abstinence [22, 23]. We first reproduced in PD rats the findings on alcohol-driven microstructural alteration in the fimbria/fornix obtained in AUD patients and alcohol-preferring msP rats [9]. Then, using MRI and quantitative immunohistochemistry, we found an associated reduction in myelin content in this fiber tract. Using multi-site electrophysiological recordings, we were able to demonstrate that this alteration was associated to impaired effective connectivity form the HC to the PFC. We hypothesized that during abstinence, the interference in the HC-PFC communication impedes the extinction of maladaptive memories, affecting behavioral flexibility. Importantly, in a recent study we demonstrated reduced behavioral flexibility in PD rats [24]. We introduce a simple quantitative neural network model to make these ideas more specific and concrete. Finally, searching for translational evidence of the above findings, we used an unpublished dataset on concomitant dw-MRI and cognitive measures in AUD patients, and discovered an association between the magnitude of microstructural alteration in the fimbria/fornix and the reduction in cognitive flexibility.

## Materials and methods

### Human study

The participants were 83 men enrolled in two different groups: a cohort of 35 healthy controls and a cohort of 48 abstinent recently detoxified AUD patients (Table 1) recruited at the Central Institute of Mental Health in Mannheim, Germany (WHO-International Clinical Trials Registry Platform: DRKS00003357). Analyses of diffusion data from this trial has been recently reported [9, 25]. Here we focus on the investigation of *local* diffusion effects data and hence included a subset of participants of the previously reported sample for whom high quality dw-MRI data was available. AUD patients were scanned at 1–2 weeks of admission into the clinic. The key inclusion criteria for the AUD group were the diagnosis of an alcohol dependence according to DSM-IV (here equated to AUD), controlled abstinence of at least 2 weeks prior to the MRI session and completion of medically supervised detoxification (treatment of withdrawal symptoms with short-acting benzodiazepines had to be completed for at least 3 days), absence of severe psychiatric co-morbidities or abuse of other substances (except smoking). Descriptive statistics of demographic data and clinical descriptors appear in Table 1. Patients participated in a standardized inpatient multi-professional medically-supervised therapy schedule [26, 27]. The local ethics committee approved study procedures and all participants provided informed written consent.

**Table 1:** Demographic and clinical data for healthy controls and patients

	Control (n = 35) Mean (SD)	AUD (n = 48) Mean (SD)	Statistics	Significance
*Demographical variables*				
Age (years)	40.9 (9.8)	47.5 (10.1)	t_(81)_ = 2.957	p = 0.004*
Education (no post-secondary educ./apprenticeship only/attended college or higher)	1/20/14	4/27/17	Z = 3.766	p = 0.288
*Substance use patterns*				
Ethanol (g/day; mean of last 90 days)	6.1 (5.7)	202.5 (196.8)	t_(81)_ = 5.809	p < 0.001*
ADS (total score)	2.12 (2.4)	15.31 (6.8)	t_(79)_ = -10.744	p < 0.001*
Abstinence before MRI scan (days)	-	12.52 (7.8)	-	-
Smoking (yes/no)	3:31	32:16	Chi^2^_(1)_ = 25.495	p < 0.001*
Cigarettes per day in smokers (0–10/ 11–20/ 21–30/ >30)	1/0/2/0	2/8/14/8	Chi^2^_(6)_ = 4.162	p = 0.210
FTND in smokers (total score)	5.3 (4.6)	6.1 (2.1)	t_(33)_ = 0.558	p = 0.581
*Clinical scales*				
OCDS (total score)	1.53 (1.3)	16.85 (7.7)	t_(78)_ = -11.178	p < 0.001*
BDI	2.09 (2.6)	17.20 (11.4)	t_(81)_ = -6.144	p < 0.001*
STAI	30.62 (7.0)	45.92 (13.2)	t_(79)_ = -7.574	p < 0.001*

ADS = Alcohol Dependence Scale (missing values for N = 1 HC, N = 1 AUD Cohort); BDI = Beck Depression Inventory; FTND = Fagerstroem Test for Nicotine Dependence (missing values for N = 32 HC, N = 16 AUD); OCDS = Obsessive-Compulsive Drinking Scale (missing values for N = 3 HC, N = 0 AUD); Smoking status (missing values for N = 1 HC, N = 0 AUD); STAI = State Trait Anxiety Inventory (missing values for N = 0 HC, N = 2 AUD); SD = standard deviation; * = significant group main effect *p* < 0.05

#### Neuropsychological testing

Patients were administered a battery of executive function tests on the day of the first MRI session. The battery consists of four tasks selected based on their well-established performance deficits in AUD patients. First, the Trail Making Test (TMT) aims to test speed of processing, visual search, motor performance, sequence alternation, and cognitive flexibility [28]. The test consists of two parts (i.e., A and B, the latter being more complex) that must be performed as quickly as possible, and the direct scores are reflecting the time required to complete each task, so the lower the score, the better the cognitive performance. The TMT is the most widely used neuropsychological screening for cognitive impairment and executive deficits, and it is the only one of our battery with normative data for healthy populations and AUD patients [29]. Importantly, the performance scores from our patient sample match-up very closely with the published norm values for AUD, further supporting the validity of our clinical sample. Second, the closely related Number Symbol Test (NST, [30]) also measures processing speed. The score of the NST reflects the number of successful items solved in a given time (90 s), so a higher score reflects a better cognitive performance. Third, the Wisconsin Card Sorting Test (WCST), is meant to test abstract reasoning, set shifting and flexibility without a time-limiting component [31], and finally, the Stroop test evaluate cognitive interference [32]. Healthy subjects were not tested.

#### Group characteristics

Descriptive statistics of demographic data and clinical descriptors appear in Table 1.

### Animals and alcohol exposure

Forty-eight male Wistar rats, initial weight 220 to 250 g, were used in the study (Charles River). Animals were housed 2–4 per cage (Type-IV; Ehret) under a 12 hours’ light/dark cycle with *ad libitum* access to food and water. All experiments were approved by the Animal Care -and Use Committee of the Instituto de Neurociencias de Alicante, Alicante, Spain, and comply with the Spanish (law 32/2007) and European regulations (EU directive 86/609, EU decree 2001 − 486, and EU recommendation 2007/526/EC).

The chronic intermittent exposure to ethanol vapor (CIE) was performed as described previously [33]. Briefly, rats were exposed to either ethanol vapor (post-dependent rats) or normal air flow (control rats), obtaining in this way two independent groups. The alcohol was delivered by dosing pumps (Knauer) into electrically heated stainless-steel coils (60 °C) connected to an airflow of 18 L/min. Exposure was for 7 weeks, combining daily intoxication during 14 h of ethanol vapor with 10 h of withdrawal. Blood ethanol concentration (BEC) were monitored and maintained around 250–300 mg/dL. Briefly, BECs were measured twice per week, in 1 animal per cage on a rotation schedule to limit the stress of the procedure. After last exposure cycle, rats were in abstinence during 10 days before initiating experiments. This exposure protocol is known to induce long-lasting behavioral as well as molecular changes in all major domains of the addiction circuitry, i.e. in motivational [34], emotional [35] and cognitive circuits [24, 36].

### Anesthesia and animal preparation

To perform the experiments, animals weighting 400 to 500 g were intraperitoneally anesthetized with urethane (1.4 g/kg). When necessary, anesthesia was reinforced with a fifth of the initial dose to assure absence of reflexes. The experiment started with the MRI session. Animals were secured in an MRI-compatible cradle, constantly supplied with 0.8 L/m oxygen in air, with a face mask, and their temperature maintained at 37 ± 5 °C with a water heating pad. Breath distention, heart rate, blood oxygen saturation, and breathing rate were monitored (MouseOx, Starr Life Sciences, Oakmont, US). After MRI data acquisition, animals were transfer to a stereotaxic frame for the electrophysiological recordings in the HC and PFC. Physiological monitoring, temperature control and oxygen supply continued as before. At the end of the electrophysiological experiment animals were sacrificed and their brains process for histological analysis.

### Magnetic resonance imaging (MRI)

#### MRI protocol in humans

Scanning was performed with a 3 T whole-body tomograph (MAGNETOM Trio with TIM technology; Siemens, Erlangen, Germany). In order to assess the individual brain morphology of each participant, high-resolution three‐dimensional T1‐weighted anatomical images (MPRAGE) were collected with 192 contiguous sagittal slices, slice thickness = 1.0 mm; field of view = 256 × 256 mm^2^, repetition time (TR) = 2.3 s, echo time (TE) = 3.03 milliseconds, inversion time = 900 milliseconds, and flip angle = 9°. DTI data was acquired using an Echo Planar Imaging spin-echo diffusion sequence with the following parameters: TR = 4 ms, TE = 84 ms, 41 gradient orientations uniformly distributed plus one non-diffusion weighted images, b-value = 1000 s/mm^2^, matrix size = 128 × 128 × 64, and an isotropic spatial resolution of 2 mm^3^.

#### MRI protocol in rats

The experiments were carried out in a horizontal 7 T scanner with a 30 cm diameter bore (Biospec 70/30, Bruker Medical, Ettlingen, Germany). DTI data were acquired using an Echo Planar Imaging spin-echo diffusion sequence with the following parameters: TR = 8000 ms, TE = 29 ms, 30 gradient orientations with b-value = 1000 s/mm2 plus three non-diffusion weighted images, matrix size = 128 × 128 × 16, in-plane resolution = 0.225 × 0.225 mm2, slice thickness = 1 mm. Multi-compartment relaxometry data were acquired using a Multi-Slice Multi-Echo (MSME) protocol with the same geometry of the diffusion scan and the following sequence-specific parameters: TR 6000 = ms, TE varied in the range 5-155 ms in steps of 5 ms, and 2 repetitions.

#### MRI analysis in humans

Diffusion data were preprocessed to correct for Eddy current and motion distortions using affine registration. DTI analysis was done with the software ExploreDTI v.4.8.4 [37]. The pipeline included free-water correction according to [38] and the Robust Estimation of Tensors by Outlier Rejection approach [39] to exclude corrupted volumes when calculating the tensor; both approaches are needed to mitigate CSF contamination [40].

Fractional Anisotropy (FA) maps computed through the DTI models were fed into an in-house modified version of the Tract-Based Spatial Statistics (TBSS) routine of FSL19 [41], in which the normalization to MNI standard space is performed using more accurate normalization tools (ANTs package) [42]. After skeleton extraction, skeletonized maps were obtained for FA by applying the pre-computed registration and skeletonization steps. A general linear model was used within a voxel-wise, permutation-based, non-parametric statistical framework [41] to test for significant differences between alcohol and control, controlling for age and multiple comparisons across clusters using Threshold Free Cluster Enhancement. We employed 10,000 permutations, and a corrected voxel-wise p-value < 0.05 was considered statistically significant. The normalization and skeletonization routine was combined with an automatic region of interest (ROI)-based aggregation based on white matter labelling in standard space (JHU ICBM DTI 81 Atlas by Mori et al. 2008, also available in FSL, total 50 ROIs) as previously described [43]. This allows to simultaneously minimize registration- and interpolation-related biases (through skeletonization) and increase sensitivity through ROI-wise averaging [44]. After skeleton extraction, the skeleton was combined with the ICBM DTI 81 Atlas, and for all participants, mean FA values were calculated in each ROI belonging to the white matter parcellation. Two-sample t-tests were performed in each ROI to test for significant differences in AUD versus control, and the p-value was corrected for multiple comparisons using a false discovery rate approach.

In order to compare the effect size, we calculated in ROIs with significant differences across conditions, the percent change in FA according to the following formula:

ΔFA=(< FA_2_>-<FA_1_>)/<FA_1_>

Where 1 stand for healthy condition and 2 for pathology condition.

For the association with cognitive variables, we first identified those that correlated with FA in the fimbria/fornix by Pearson correlation and then, given the highly significant association between them (the individual who performs well on one test, performs well on the rest), we obtain a single cognitive component through principal component analysis (PCA). The first component explained most of the variance in the cognitive tests (PC1 = 87%). Then, considering the inherent influence of age both on microstructural status of the brain and cognitive decline [43], we performed a partial Kendall tau correlation between FA and PC1 in order to account for the influence of age on our data.

#### MRI analysis in rats

All data were preprocessed to correct for Eddy current and motion distortions using affine registration. In addition, diffusion data were non-linearly registered to MSME data to correct for EPI distortion using ANTs [45]. DTI analysis was done with the software ExploreDTI v.4.8.4 [37] as we described previously [9]. Multi-compartment relaxometry data were fitted to a bi-exponential decay (representing water trapped into myelin sheets and intra-extra cellular water) using in-house Matlab (R2015b, The Mathworks, Inc., Natick, MA) code, and the fraction of the signal associated to the fast-relaxing component was interpreted as a proxy for myelin content [46].

From the preprocessed data, the following parameter maps were computed for each subject: FA and myelin fraction (MF). Whole brain tractography was calculated in native space using a deterministic, tensor-based approach; tract termination criteria were FA < 0.15 and angle > 30 degrees. The fimbria was then manually reconstructed in each rat using endpoints placed in anatomically plausible positions. Average values of FA and MF and tract volume in pixels were calculated in the fimbria for each rat.

### In vivo electrophysiology

Surgical and stereotaxic procedures were performed as described previously [47]. Two concentric bipolar stimulating electrodes (WPI, London UK) were used to stimulate the medial perforant pathway (from lambda: AP: 0; ML: 4.1; DV: 2.3–2.7 mm) and the fimbria (from bregma AP: -1.5; ML: 0.4; DV: 3,2 mm), according to [48]. Two multisite silicon probes (single shank, 32 channels, 100 μm spacing; Neuronexus Technologies) connected to multiple high impedance head-stages were lowered into intermediate hippocampus and medial prefrontal cortex using coordinates with respect to bregma: AP -4.4; ML 2.6; DV 3.5 mm and AP 3.4; ML 0.5; DV 4.5 mm, respectively. Before brain insertion, probes were immersed in a saturated solution of DiI in ethanol, for posterior postmortem confirmation of probes placement. A silver chloride wire in contact with the neck skin worked as a ground for the recordings. Electrophysiological signals were filtered (high-pass 0.1 Hz), amplified and digitalized using Multi Channel Systems recording hardware and software (10 kHz sampling rate).

First, spontaneous activity was simultaneously acquired in the prefrontal cortex (prelimbic and infralimbic) and hippocampus (CA1 and DG) for correlation and coherence analysis. Then, evoked potentials were recorded in response to different electric stimulation protocols applied using a pulse generator and current source (STG2004, Multichannel Systems, Reutlingen, Germany). A stimulus-response curve protocol consisted in single biphasic 100 µs duration pulses at different intensities (ranging from 60 to 900 µA) delivered every 15 s, with each intensity presented four times. Second, a paired-pulse protocol with two pulses of suprathreshold and identical intensities applied at varying inter-pulse time intervals (from 10 to 80 ms) was used to investigate feed-back inhibition (around the time of maximal GABA_A_ conductance) and facilitation (around the time of maximal GABA_B_ conductance) [49]. Finally, a long-term potentiation protocol (LTP) consisting in six trains of pulses of 400 Hz lasting 20 ms, delivered at a 10 s interval, and repeated six times at an interval of 2 min, was used to investigate long-term synaptic plasticity (16 min of duration).

Neuronal firing in response to the stimulation protocols was quantified as the amplitude of the population spikes (PS) recorded in the DG and CA1 soma layers, respectively. Similarly, the synaptic activity evoked by stimuli was measured as the slope of the evoked postsynaptic potential (EPSP) recorded in the DG molecular layer and the CA1 *stratum radiatum*. The effect of pair-pulse stimulation was measured as the ration of the second PS (PS2) divided by the first (PS1) and that of LTP as the percentage increase in the PS after LTP induction vs. the baseline. Activity propagation from the HC to the PFC was computed as the amplitude of the evoked potential in the PFC divided by the simultaneously recorded PS in the CA1.

### Tissue processing for histology and immunohistochemistry for myelin basic protein

After completion of each experiment, anesthetized-rats were intracardially perfused with 100 mL of 1% phosphate-buffered saline (PBS) solution followed by 50 mL of cold 4% paraformaldehyde (PFA) in PBS. Brains were kept for 24 h on 4% PFA post-fixation at 4° and prepared for coronal Sect. (50 μm) using a vibratome (VT1000S, Leica Microsystems). Slices were collected into 24-well plates containing 1% PBS. For each rat, tissue sections were selected for the fimbria, between 2- and 3-mm posterior to Bregma. Then slices were kept in a solution of sodium citrate (pH 6.0) and introduced in a thermomixer (Eppendorf Ibérica) during 20 min once the temperature has reached 80 °C. The brain slices from all animals, experimental and controls, were processed and stained simultaneously, to assure equal conditions. After rinsing with 1% PBS, sections were incubated with a blocking solution [10% goat serum donor herd + 4% bovine serum albumin in PBS supplemented with 0.5% Triton X-100] for 2 h. Sections were then incubated with mouse anti-MBP antibody (1:250, Merk Millipore, Darmstadt, Germany) in the blocking solution overnight at 4 °C. After rinsing again in PBS, sections were incubated for 90 min with Alexa-488-conjugated goat antibody (anti-mouse, Termofisher, 1:500), rinsed in PBS and mounted for photography under a fluorescence light microscope (Neurolucida, MBF Bioscience, Netherlands). Image acquisition and quantification was done blind for the experimenter. Images were then analyzed with the ImageJ program (National Institute of Health, USA) (Schindelin et al., 2012). Two ROIs per fimbria were taken from two consecutive sections from both, right and left hemispheres, per animal. Mean intensity in each ROIs was averaged per animal. Intensity of fluorescence was determined considering the area of the region selected (Integrated density = mean fluorescence * area) and the intensity of the background. In this way, we obtained the corrected total fluorescence (CTF) with this formula: CTF = Integrated density – (area * mean background fluorescence).

### Statistical analyses in the animal experiment

Experimenters were blind to the animal’s experimental group until the completion of individual data collection and analysis.

All statistical analyses were conducted in SPSS (IBM Corp. Released 2012. IBM SPSS Statistics for Windows, Version 21.0. Armonk, NY: IBM) or GraphPad Prism version 7.00 for Windows, (GraphPad Software, La Jolla, CA, USA). Data are expressed as mean ± SEM. All animals but 4, which died during the electrophysiological surgery, were included in the analysis. For DTI analyses, the dependent variables were the parameters described previously: FA, MD and MF. Applying the ROUT module set at 1, we found four outliers for MD and one for MF. Then, unpaired one-tailed t-tests were applied to test for significant differences across conditions (alcohol exposed and controls), based on the premise that alcohol consumption affects axonal and myelin integrity [9].

For electrophysiological analyses, criteria for inclusion in final analysis were correct location of recording and stimulating electrodes (all remaining animals were included). For the evoked potentials the dependent variables were the amplitude of the evoked potential in the PFC, the PS and EPSPs recorded in CA1 and DG, the PPRs, the percentage of potentiation after LTP, and the propagation index. These variables were analyzed with unpaired two-tailed t-tests or ANOVAs, applying Bonferroni’s *post hoc* tests when significant effects were obtained. For the spontaneous activity, spectral coherence and broad-band cross-correlations were computed, corrected with a surrogate analysis for intraindividual significance and compared across groups with an unpaired two-tailed t-test.

In the histological analyses, three outliers were found applying the ROUT module set at 1. Then, an unpaired two-tailed t-test was applied. All distributions were checked to be normal using D’Agostino-Pearson omnibus normality test, and a *P-*value < 0.05 was considered statistically significant.

### Computational model

#### Network construction and learning rules

We used a computational model based on the TraceLink model [50] with some modifications (see below). Most parameters were kept as in the original model as they have proven validity to account for a wide range of biological observations, such as retrograde and anterograde amnesia or a state of permastore [50]. Briefly, the model consisted of two layers, simulating hippocampal and prefrontal learning dynamics. Each layer was composed by nodes that simulate groups of neurons and that could take values of ‘1’ (when active) or ‘0’ (when inactive). The hippocampal layer was composed by 42 nodes and the prefrontal layer by 200 nodes to account for the difference in memory capacity between the two brain areas. Nodes were connected within and between layers with 50% of probability and with excitatory synapses implementing Hebbian plasticity. A simulated competitive mechanism allowed only k nodes, those that received the highest input, to be active in each layer in each cycle of activity simulation. The total input to a node is calculated as the weighted sum of all inputs received from connected nodes plus a noise level (randomly taken from a uniform distribution function between 0 and 0.5). We set k to 7 in the hippocampus and to 10 in the prefrontal cortex. The model simulated the processes of acquiring, consolidating and recalling of memories (specific activity patterns), which includes memory extinction as new patterns are learned. A pattern consisted of 7 and 10 active nodes in the hippocampal and prefrontal layers, respectively, in agreement with the established parameter k [50]. We performed 10 blocks of 20 simulations each.

To test the role of the hippocampal-prefrontal connectivity in memory extinction, random patterns were generated and presented to the network for learning and later recall. In average, every two patterns shared an overlap of ~ 1/6 in the hippocampus and ~ 1/20 in the prefrontal cortex. The presentation of a pattern to the network produced the updating of the synaptic weights (acquisition phase) following a Hebbian learning rule that included synaptic potentiation and depression terms:

1 $$\varDelta {\omega }_{ij}={\lambda }_{+}{a}_{i}{a}_{j}-{\lambda }_{-}{a}_{i}(1-{a}_{j})$$

where *Δω*_*ij*_ refers to the variation of connection strength (*ω*) between post-synaptic node *i* and pre-synaptic node *j*, *a*_*i*_ and *a*_*j*_ refers to the state (‘1’ or ‘0’) of node *i* and *j*, respectively, and *λ*_*+*_ and *λ*_*−*_ are the learning rates for “encoding” and “forgetting”, respectively. “Encoding” refers to the process of learning patterns through strengthening connections in the network while “forgetting” refers to the process of unlearning previously strengthened connections. The values of *ω* were kept between 0 and 1. During acquisition, the value of *λ*_*+*_ is set to 0.4 for between layers and within hippocampus connections and to 0.06 for within prefrontal cortex connections, reflecting on the dominant associative role of the hippocampus during the initial stage of memory formation. Unless otherwise specified, the value of *λ*_*−*_ was set as 75% of the value of *λ*_*+*_ for all connections.

After memory acquisition, a consolidation phase followed. In this phase, a randomly chosen pattern, from the already learned patterns, was presented to the network and the network activity was updated during 8 cycles while the strength of the synapses within the prefrontal cortex layer were modified following Eq. (1). Note that in each cycle, the pattern to consolidate could be different, i.e. the dynamics of the network chose which pattern to consolidate in each cycle. In the consolidation phase, the learning rate *λ*_*+*_ was set to 0.0025 for the within prefrontal layer connections and to 0 for all other connection and *λ*_*−*_ was set to 75% of these values. This consolidation procedure was repeated 3 times after each acquisition phase [50].

#### Network model for the AUD condition and memory testing

To mimic the AUD findings, the effective connectivity between the HC and the PFC was decreased by reducing a percentage (from 0 to 100% in 20% steps) of randomly selected synapses from the HC to the PFC. The network acquired and consolidated 10 patterns before “lesioning” the HC-PFC connectivity, and then 10 more patterns were acquired and consolidated. We tested the recalling capabilities of the network by presenting 50% of a learned pattern (cue) to the prefrontal cortex layer and by allowing the dynamics of the model to complete and recover the rest of the pattern. With this procedure, we tried to simulate a situation in which a specific cue retrieves information stored in memory, like alcohol-associated cues retrieve consumption memories. We estimated the recall accuracy of the model by computing a recall score that was obtained by calculating the ratio between the number of nodes that were active in the prefrontal cortex and were part of the original pattern but not of the cued pattern, and the number of total nodes of the pattern that were not in the cue. Chance was estimated by computing the recall score for a random pattern that was not previously learned. The proportion of consolidation of new patterns (those encoded after HC-PFC “lesion”) was calculated as the number of new patterns (patterns from 11 to 20) that were consolidated after HC-PFC manipulation over the total number of consolidated patterns.

Finally, to investigate whether new acquired memories (new patterns) could replace previously learned ones (old patterns) when they shared a considerable amount of information, we created pairs of patterns with a 50% overlap and tested the flexibility of the network, i.e. the capacity of the network to replace old information with new one, for different values of “forgetting” in the learning rule and different levels of hippocampal-prefrontal impairment. With this procedure, we wanted to simulate those cases in which the environmental contingencies have changed and a previously learned association needs to be updated to be adaptative. We used the same protocol for the acquisition and consolidation of patterns as before and differed only on how the patterns were created. In this case, the first 10 patterns presented to the network were randomly created as before, but the subsequent 10 patterns shared 50% of overlap in the prefrontal cortex with previously learned patterns. Thus, every two patterns shared 50% of nodes in the prefrontal cortex. During recall, the network was tested using the 50% of overlap as cue. Flexibility was computed as:

$$Flexibility= \frac{{N}_{new}-{N}_{old}}{{N}_{new}+{N}_{old}}$$

where N_new_ and N_old_ are the number of cases in which the new or the old pattern, respectively, were recalled. We considered successful recall when the recall score (see above) was equal or greater than 0.75 for both, the new or the old pattern. A flexibility value of 1 indicated total flexibility, with only new patterns being recalled, a value of 0 indicated equal recall for old and new patterns and a value of -1 indicated that only old patterns were recalled.

## Results

### Fimbria/fornix microstructure is impaired in PD rats during early abstinence

**Fig. 1:**
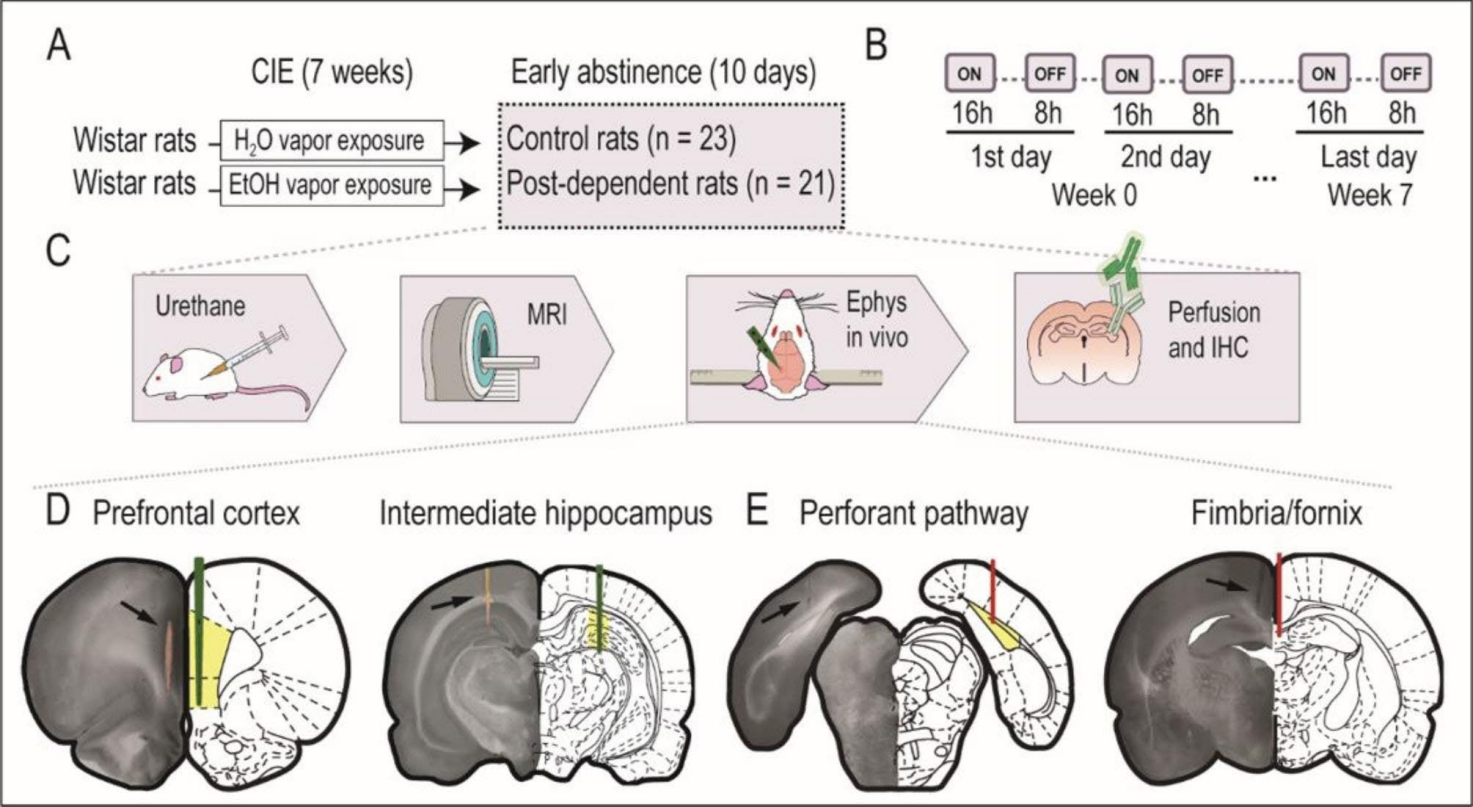
Experimental design of the animal study. (**A, B**) Schematic representation of the protocol used to prepare postdependent animals and controls, including the chronic intermittent exposure (CIE) regime, respectively. (**C**) Schematic representation of the sequential procedures applied to each subject in the study. (**D**) Location of the recording (green) and stimulating (red) electrodes, respectively. Histological image (left) and corresponding atlas section (right) [48]. The yellow shading marks the targeted region. Final electrode positioning was always based on the recording of well-known pathway-specific evoked potentials. Arrows point to the DiI trace left by the recording electrode and the tissue damage produced by stimulating electrodes

**Fig. 2:**
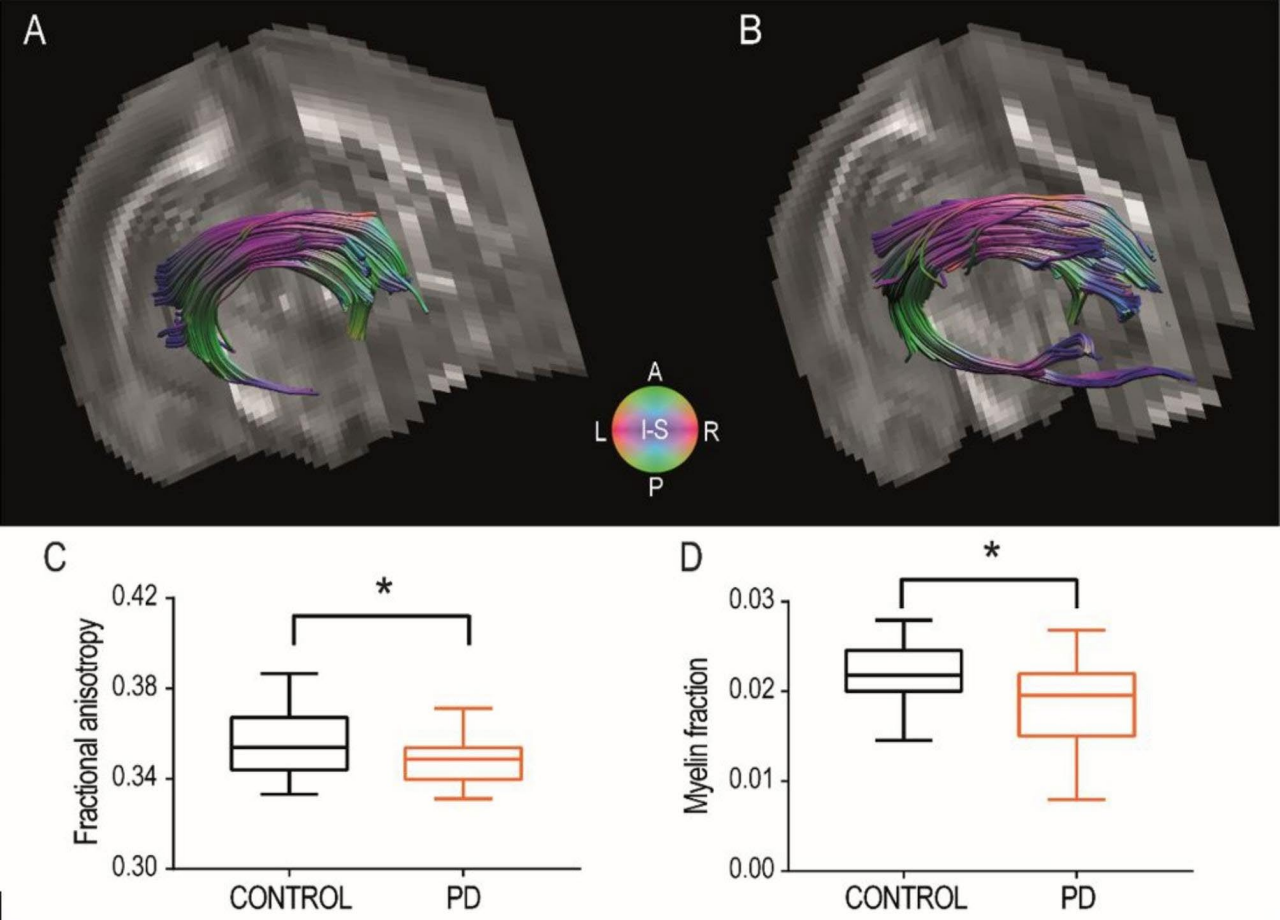
Effects of chronic alcohol exposure in dw-MRI parameters measured in the fimbria/fornix. (**A, B**) Representative DTI reconstruction of the fimbria/fornix in native space for one control rat (**A**) and one post-dependent rat (**B**). Both representations are displayed using DTI color conventions, superimposed on the FA maps. (**C, D).** Group-level values of fractional anisotropy (n = 16 control rats; 15 PD rats) (**C**) and myelin fraction (n = 15 control rats; 15 PD rats) (**D**) in the fimbria/fornix are shown for control and PD rats. Box-and-whisker plot showing the first and third quartile (box) and min/max values (whiskers). The horizontal line in the box represents the median. The asterisk represents significant difference in the unpaired t-test statistic (*p < 0.05)

We first investigated the microstructural integrity of the fimbria/fornix in PD rats comparing with age-matched controls (Figs. 1 and 2A-B). We found significantly lower FA in PD rats (Fig. 2C; unpaired t-test, t(29) = 1.88, p = 0.035), consistent with axonal damage [51]. We also found that the myelin fraction, measured as the MSME signal fraction associated to the shorter relaxation time, was significantly smaller in alcohol abstinent animals (Fig. 2D; unpaired t-test, t(28) = 1.90, p = 0,033), suggesting demyelination as the underlying mechanism.

### Myelin basic protein content in fimbria/fornix is lower in early alcohol abstinence

**Fig. 3:**
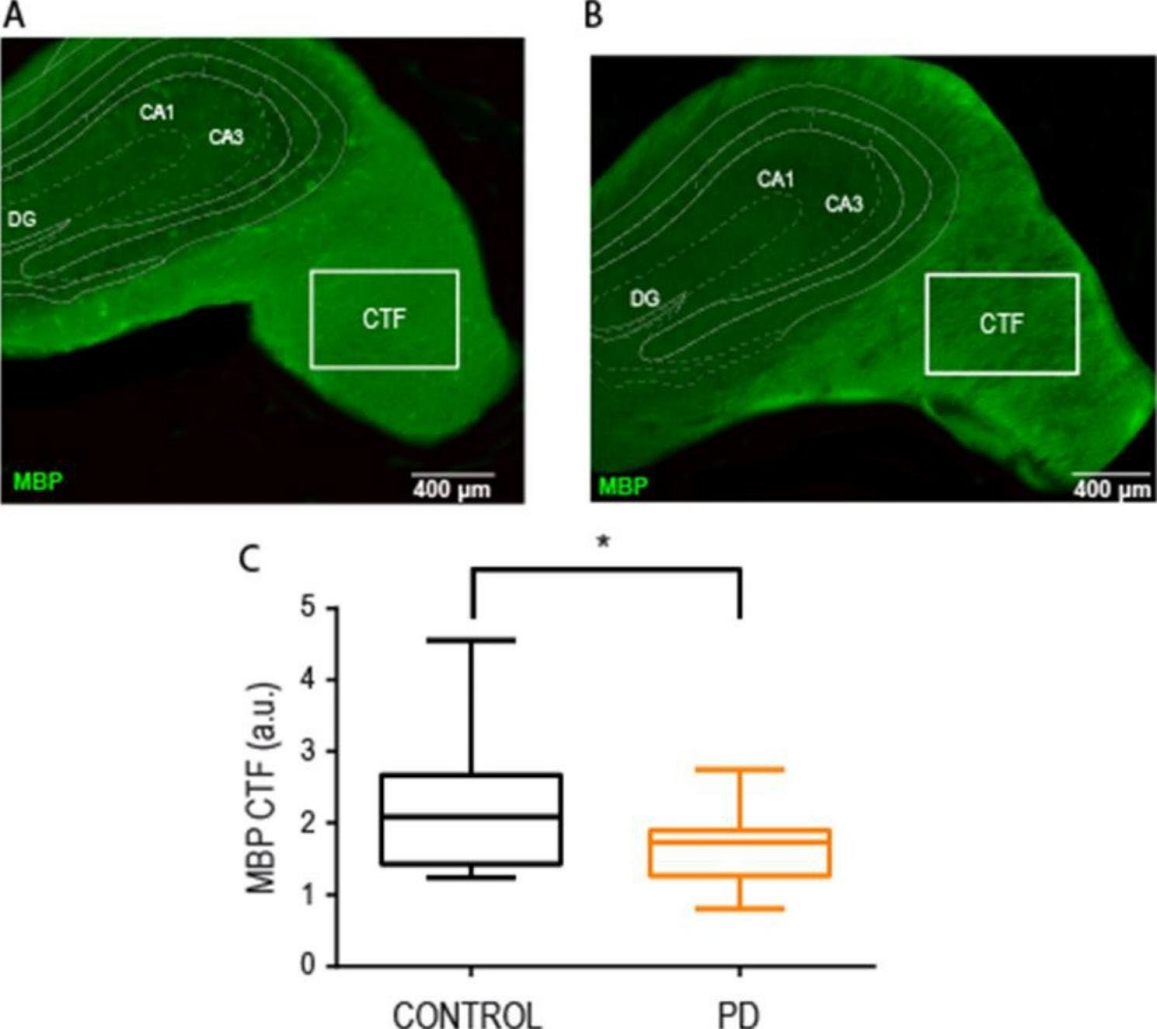
CIE-induced alteration of the MBP amount in the fimbria tract. (**A, B**) Representative section of the fimbria of a control rat (**A**) and a PD rat (**B**) labeled for MBP (green) to assess white matter integrity. (**C**) Quantification of the MBP corrected total fluorescence (CTF, see methods) in 19 control and 17 PD rats and expressed in arbitrary units (a.u.). Box-and-whisker plot showing the first and third quartile (box) and min/max values (whiskers). The horizontal line in the box represents the median. The asterisk represents significant difference in the unpaired t-test statistic (*p < 0.05)

We investigated white matter structure using quantitative immunohistochemistry in the same animals. Myelin basic protein (MBP) is a component of the myelin sheath commonly used to quantify, based on its staining intensity, the integrity of the white matter [52]. We immunostained MBP in the fimbria/fornix of control and PD rats (Fig. 3A-B, respectively) and quantified its content as fluorescence staining intensity. We found significantly lower levels of MBP in the PD rats at two weeks of abstinence compared to the control animals (Fig. 3C; unpaired t-test, t(34) = 2.469, p = 0.018). This result confirms the DTI finding and the hypothesis of an alcohol driven demyelination in early abstinence.

### Chronic intermittent alcohol exposure increased hippocampal excitability

**Fig. 4:**
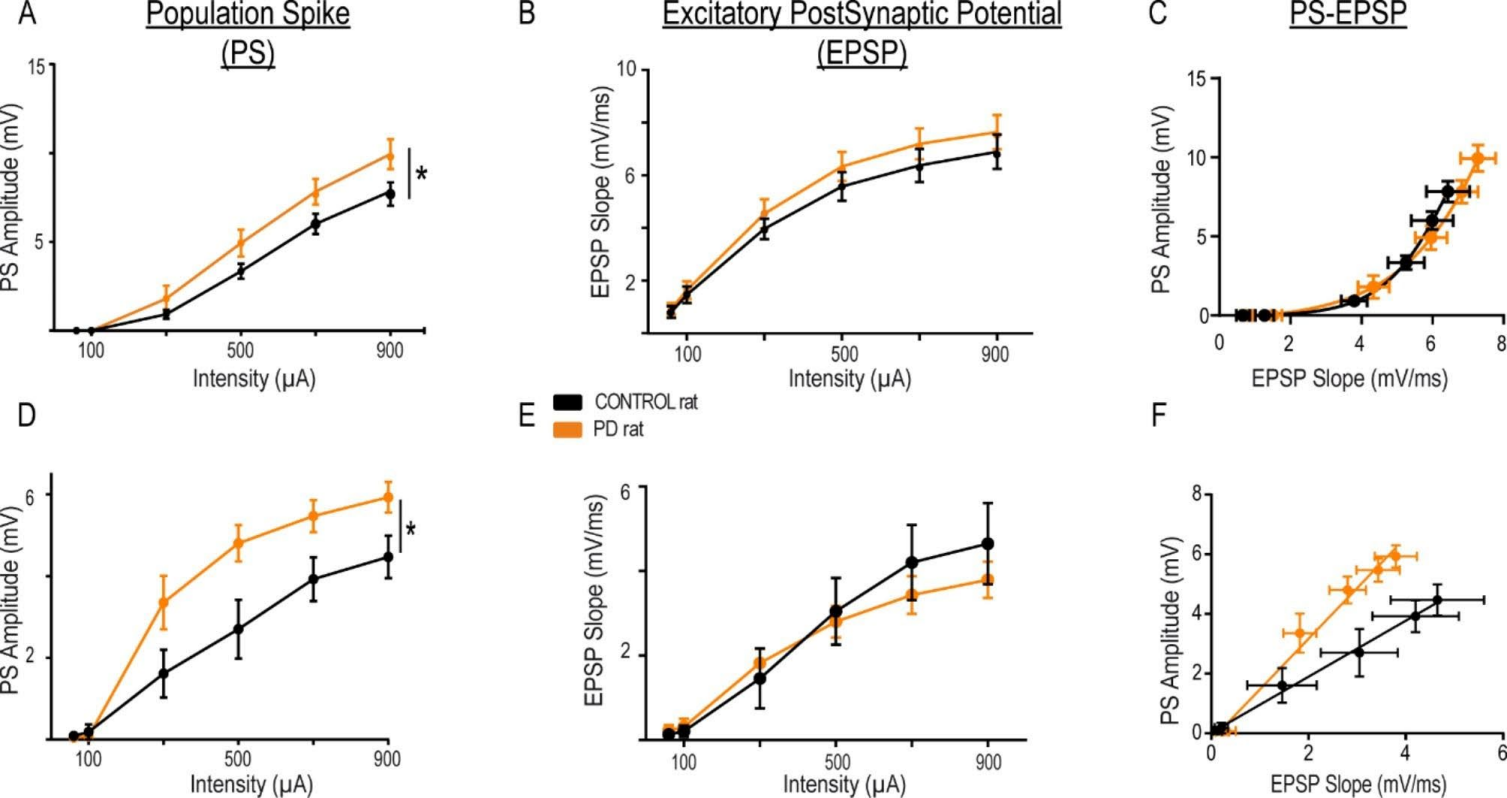
Chronic intermittent ethanol exposure increased hippocampal excitability. **A, B.** PS amplitudes and EPSP slopes, respectively, recorded in the DG in response to perforant path stimulation of increasing intensities in PD (orange) and control (black) rats. **C**. Input (EPSP) - output (PS) curves in the DG. Data was fitted with a logarithmic regression. No differences were found between both curves. **D, E.** Same as A, B but recorded in the CA1 in response to fimbria stimulation. **F.** Same as C for the CA1 responses. Data are fitted with linear regressions. Both curves are significantly different (extra sum-of-squares F test: F_(2,8)_ = 69,87, p-value < 0,0001). The asterisk represents significant difference in the two-way repeated-measures ANOVA for PD vs. control groups (*p < 0.05)

We next investigated the synaptic transmission and plasticity in the hippocampus, carrying out multiple in vivo electrophysiological recordings using 32-channel electrodes implanted in the hippocampus and spanning the dentate gyrus (DG) and the CA1 region (Fig. 1D). Electrical field potentials evoked by stimulation of the perforant pathway or the fimbria (Fig. 4), respectively, demonstrated facilitated principal cell firing in both regions, as measured by the larger amplitude of the population spike (PS) in abstinent PD rats vs. controls (Fig. 4A: two-way repeated-measures ANOVA group effect F(1,34) = 4.151, p = 0.049, stimulation intensity effect F(5,170) = 167, p < 0.0001, interaction F(5,170) = 2.49, p = 0.033; and Fig. 4D: two-way repeated-measures ANOVA group effect F(1,19) = 6,779, p = 0.0174, intensity effect F(5,95) = 114,6, p < 0.0001, interaction F(5,95) = 5.48, p = 0.0002) with comparable excitatory postsynaptic potentials (EPSPs) in both experimental groups (Fig. 4B, E and p > 0.05 for group effect). A regression analysis assessing the relationship between the EPSP slope and PS amplitude demonstrated an altered balance in the CA1 region (Fig. 4C and F) supporting enhanced neuronal excitability, rather than enhanced synaptic activity, in the abstinent condition.

Increased neuronal excitability can be the result of a change in the intrinsic electrophysiological properties of neurons [53], or a decrease in the inhibitory tone [54]. We used paired-pulse stimulation protocols to investigate the inhibitory tone in PD animals. In a stimulation pair at 20 ms inter-stimulus interval (ISI), the second synaptic input occurs at the maximal GABA_A_ conductance driven by the firing of interneurons recruited by the first pulse. The depression of the response to the second pulse relative to the first (Suppl. Figure 1 A), is thus proportional to the GABA_A_-mediated inhibitory tone [55]. For longer delays, in the range of 50 to 100 ms, a facilitation of the response to the second pulse dominates (Suppl. Figure 1 A), which corresponds to a disinhibitory effect mediated by presynaptic GABA_B_ receptors [49]. As shown in Suppl. Figure 1A, we found no statistically significant differences between abstinent PD rats and controls neither for synaptic depression (10–20 ms inter-pulse interval) nor facilitation (50–80 ms), suggesting that the inhibitory tone in the hippocampus was intact during abstinence in this animal model (two-way repeated-measures ANOVA F(1,24) = 0.003, p = 0.950, latency F(7,168) = 42.04, p < 0.0001, interaction F(7,168) = 0.476, p = 0.850).

Finally, we tested whether long term synaptic plasticity in the hippocampus was affected by a history of chronic intermittent alcohol drinking in the PD rats. We applied a high frequency stimulation protocol known to induce long-term potentiation (LTP) in the perforant pathway [56] and measured the amplitude of the PS (Suppl. Figure 1B,D) and slope of the EPSP (Suppl. Figure 1 C) in the DG before and one hour after LTP induction. We found that LTP in PD animals is indistinguishable from that found in control animals (PS: two-way repeated-measures ANOVA F(1,8) = 0.001, p = 0.969, intensity F(5,40) = 35.76, p < 0.0001, interaction F(5,40) = 0.252, p = 0.936; EPSP: two-way repeated-measures ANOVA F(1,8) = 0.613, p = 0.456, intensity F(5,40) = 28.4, p < 0.0001, interaction F(5,40) = 0.149, p = 0.979). Overall, we can conclude that the inhibitory tone and long-term synaptic plasticity in the hippocampus are not affected during early abstinence in PD rats.

### Effective connectivity from the HC to the PFC is lower during alcohol abstinence

**Fig. 5:**
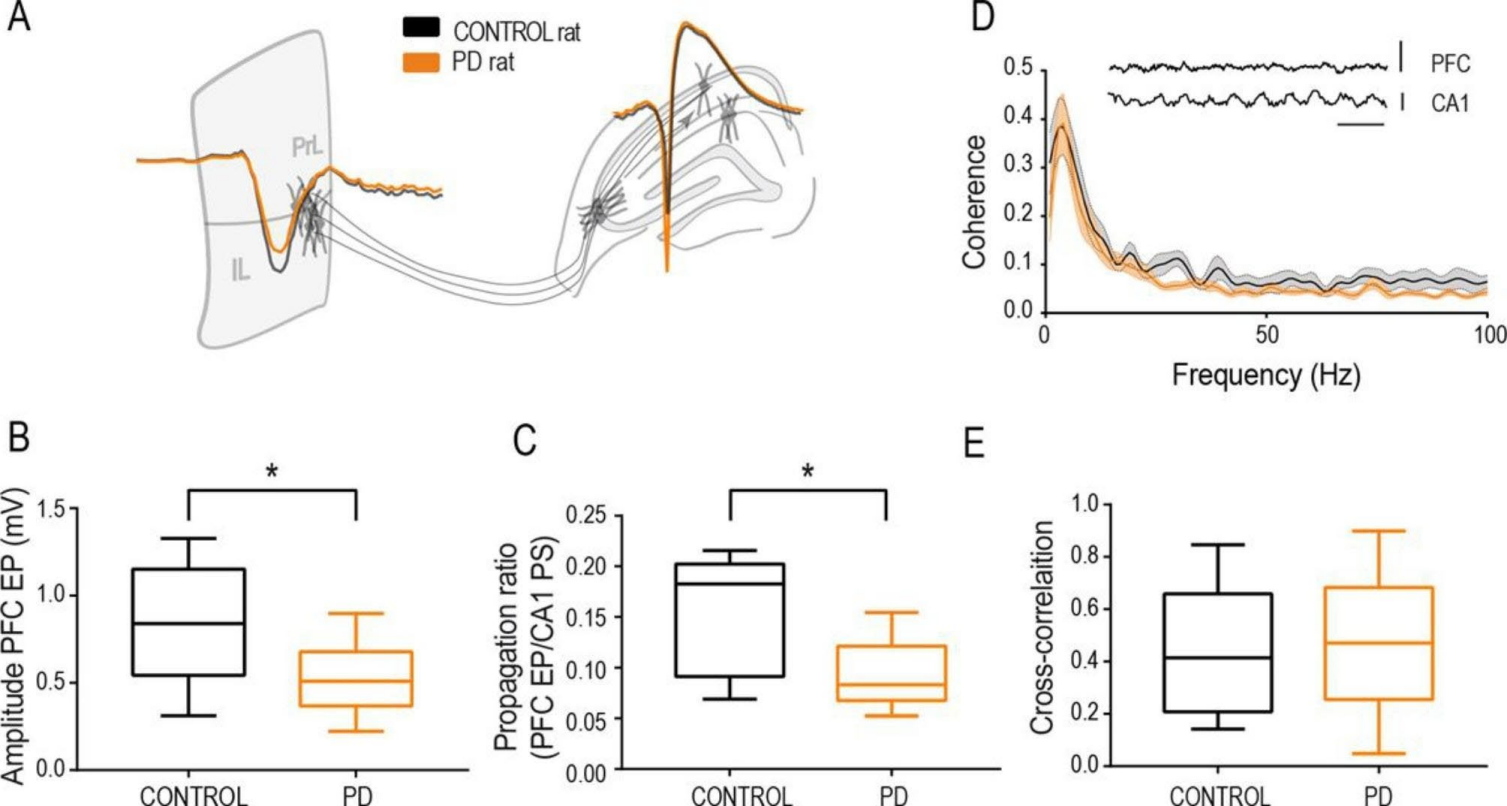
Functional connectivity from the HC to the PFC is impaired in PD abstinent rats as compared to controls. (**A**) Representative evoked potentials in the PFC and PS in CA1 in response to stimulation in control (black) and PD (orange) animals, overlaid on a schematic representation of the respective structures. (**B**) Group level quantification of the amplitude of the evoked potentials in the PFC from control and PD rats. (**C**) Effective connectivity between the HC and PFC measured as the evoked response in the PFC divided by the corresponding CA1 PS amplitude (input/output ratio). Box-and-whisker plot showing the first and third quartile (box) and min/max values (whiskers). The horizontal line in the box represents the median. Asterisks represents significant difference in the unpaired t-test statistic (*p < 0.05, **p < 0.01). (D-E) Spectral coherence (**D**) and broad band cross-correlation analysis (**E**) between CA1 and PFC spontaneous recordings in 19 control (black) and 18 PD (orange) rats. The inset shows representative spontaneous LFP traces recorded in CA1 (pyramidal layer) and PFC (prelimbic region). Scale bar in spontaneous LFPs: 1mV, 0.5ms

Finally, we investigated the effective connectivity from the HC (CA1 region) to the PFC (prelimbic and infralimbic) using simultaneous electrophysiological recordings (Fig. 1D). To do that, we electrically stimulated the most dorsal part of the hippocampal commissure, where efferences from the CA3 region of the hippocampus travel [57] and recorded the evoked potentials in CA1 and the PFC. Firing of CA3 neurons reaches the PFC through a polysynaptic activation chain involving CA1 [57, 58] (Fig. 5A). A first analysis comparing the amplitude of the PFC evoked potentials showed a significant difference, with larger potentials evoked in control animals (Fig. 5B, unpaired t-test, t(21) = 2.344, p = 0,003). Then, we quantified the functional coupling between the HC activation and the PFC response, as the propagation ratio obtained by dividing the amplitude of the PFC response by the corresponding amplitude of the CA1 PS. The results demonstrated a reduced propagation ratio in the abstinent rats (Fig. 5C, unpaired t-test, t(13) = 2.721, p = 0.01). Similar results were obtained with stimulation in the perforant pathway, in this case with a train of pulses at 10 Hz to facilitate propagation (data not shown). This last finding excluded potential unspecific contributions by a particular electrode location, and overall demonstrated a lower HC◊PFC effective connectivity in PD animals. No differences were found in the coherence (Fig. 5D) or correlation (Fig. 5E) between spontaneous local field potentials (LFPs) recorded in CA1 and the PFC (Fig. 5D, inset).

### Decreasing HC-PFC connectivity reduces flexibility in a computational memory model

**Fig. 6:**
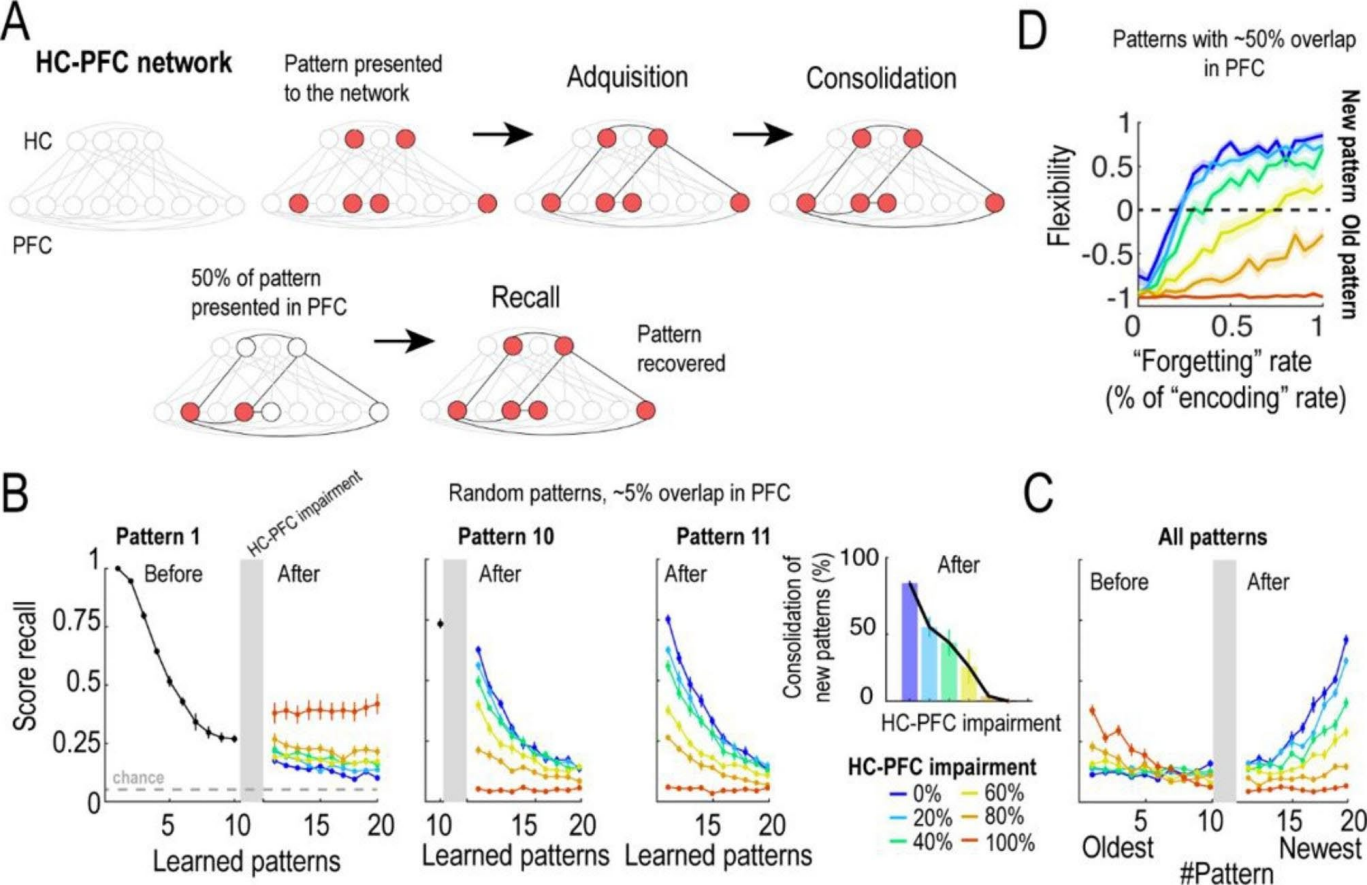
Computational model of hippocampus-prefrontal cortex for memory acquisition, consolidation and recall. (**A**) Scheme of the computational model. The model consisted of 2 layers with connections within and between them. New patterns were learned through an acquisition phase and a subsequent consolidation phase, in which the strength of the connections was modified. Recall was tested by presenting the 50% of a learned pattern in the PFC layer and comparing the retrieved pattern with the one in memory. (**B**) *Left panel*, Score recall for the first pattern learned by the model as new patterns were learned before and after manipulating the connections from HC to PFC by randomly eliminating connections (from 0–100% in steps of 20%). Horizontal dashed gray line indicates the chance level for recall. *Middle panel*, Score recall for the last pattern learned before manipulation of the HC-PFC connections (pattern 10). Same conventions as in Left panel. *Right panel*, Score recall for the first pattern learned after manipulation of the HC-PFC connections (pattern 11). Same conventions as in Left and Middle panels. *Inset panel*, Proportion of new patterns (patterns from 11 to 20) that were consolidated after HC-PFC impairment and for different values of it. Error bars indicate SEM. (**C**) Score recall for all patterns learned by the model, after all of them had been learned. Same conventions as in (**B**) (**D**) Proportion of recall of new patterns vs. old patterns for different values of “forgetting” learning rate and HC-PFC impairment when the model learned patterns that shared a 50% of overlap. Shaded areas indicate SEM.

We explored the implications of the lower HC-PFC connectivity found in the PD rats for memory consolidation, retrieval, extinction, and update by a simple computational network model. The model consisted of 2 layers of nodes endowed with Hebbian synapses that simulated learning dynamics in the HC and PFC (Fig. 6A; see Material and Methods). The network was stimulated with different activation patterns and, in an initial encoding phase, connections between nodes co-activated by the pattern within the HC and between HC and PFC layers were rapidly reinforced, generating a memory trace. Then, a consolidation phase followed in which several cycles of random pattern reactivation in the HC and PFC strengthen the links within the PFC of previously co-activated nodes, rendering endurable memories. In the retrieval phase, partial activation of successfully consolidated PFC patterns (cue), recovers the complete encoded pattern in the network (Fig. 6A). We introduced the changes in the efficiency of the HC-PFC connectivity found in alcohol abstinence by reducing the number of connections from HC to PFC.

We first studied the effect of manipulating the efficiency in the HC-PFC connection on memory extinction. We computed the recall accuracy of a consolidated pattern (pattern 1) as new patterns were sequentially learnt (patterns 2–20, Fig. 6B). The recall score of pattern 1 decayed as new patterns were learned, as an indicative of memory extinction (Fig. 6B). However, when we introduced different levels of HC-PFC impairment in the middle of the learning process (between patterns 10 and 11, Fig. 6B color lines), recall scores behaved differently. When a pattern was learned long before impairment (pattern 1), recall accuracy was increased by HC-PFC impairment. When the pattern tested for recall was the last one learned before HC-PFC impairment (pattern 10) or the one learned after it or later (patterns 11 to 20), the higher the level of HC-PFC impairment, the lower was the recall accuracy (Fig. 6B-middle and Fig. 6B-right panels). Both results together indicated an impairment in memory updating, enhancing the recall of old memories by impeding the formation of the new ones. This conclusion was supported by the decreased proportion of new patterns (11 to 20) consolidated after HC-PFC impairment (Fig. 6B-inset) and the inverse evolution of the recall score for old vs. new patterns computed after the whole set of 20 patterns was learned (Fig. 6C).

In the previous simulations the overlap between the randomly generated patterns to be encoded was low (∼5% in the PFC), but one might ask whether the network could replace old memories with new ones when the overlap between two memories is higher, since the overlap could enhance “forgetting” in the network (see Materials and Methods). This possibility might be relevant for memory updating in conditions in which the contingencies have changed (i.e. alcohol consumption is not reinforcing anymore) but memory associated cues are largely overlapping (cue reactivation). To test this hypothesis, we generated activity patterns so that old and new patterns overlapped by 50%. We also varied the rate of “forgetting” in the network to study whether a particular rate could compensate for the loss of HC-PFC efficiency. Then, we estimated how well the network replaced old with new memories by calculating a flexibility index that reflected the preference to recover new over old patterns when stimulated with the same cue (see Materials and Methods). Figure 6 C shows that, as expected, memory flexibility increased with the rate of “forgetting” but, interestingly, it was progressively impaired by decreasing levels of HC-PFC communication, supporting the fundamental role of the HC-PFC connectivity in memory flexibility, and providing a potential mechanism for the development of maladaptive and recurrent behavioral responses in AUD based on the fimbria/fornix microstructural vulnerability.

### Fimbria/fornix damage in AUD patients correlates with cognitive alterations

**Fig. 7:**
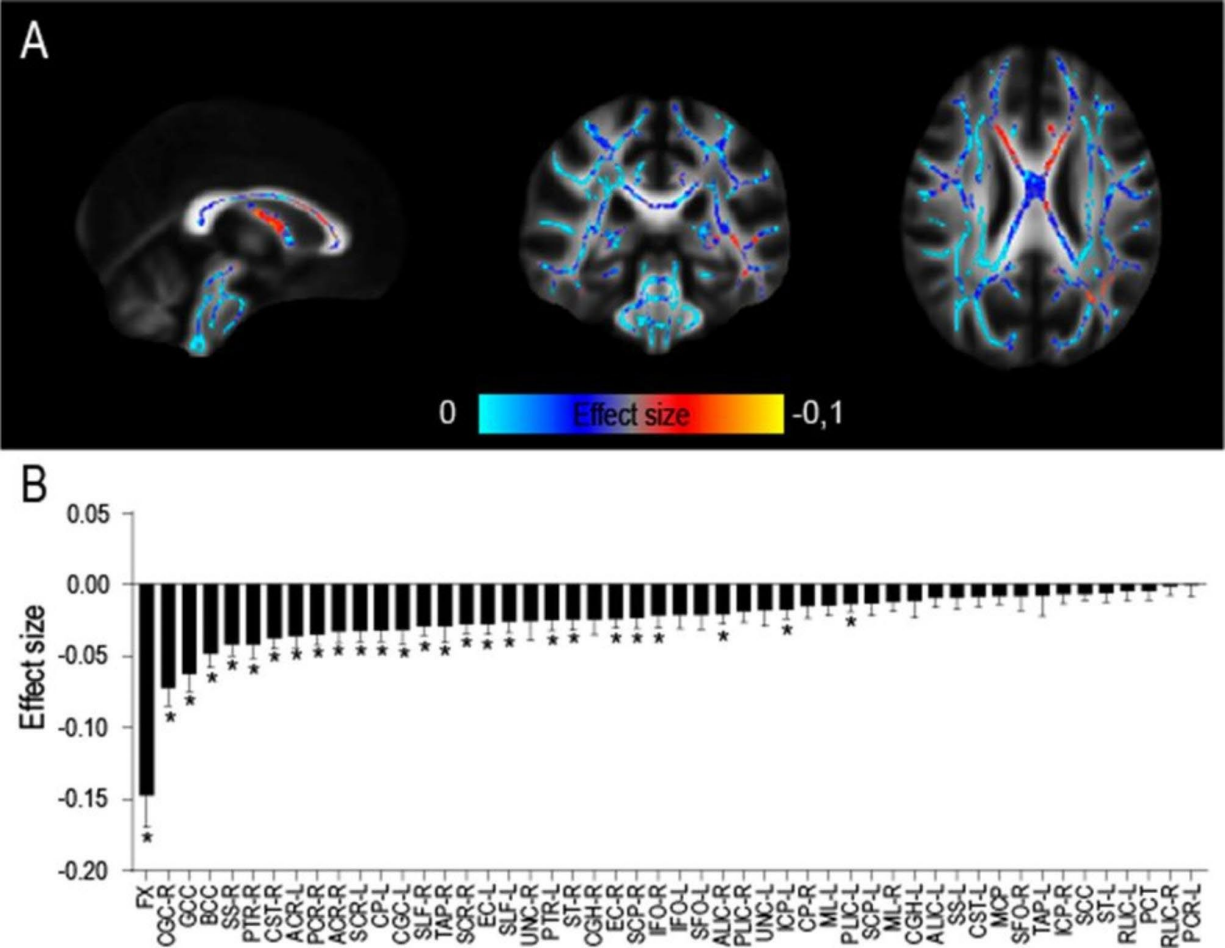
Effect size of FA alterations in AUD patients. (**A**) Effect size of age-controlled FA differences between 48 AUD and 35 controls, in ROIs where a significant difference (p < 0.05 corrected) between the two groups exists. (**B**) Histogram (mean and standard error) of effect size across all the 50 ROIs of the ICBM DTI 81 Atlas. The asterisk represents significant difference in effect size between groups (*p < 0.05). A list of abbreviations can be found in Suppl. Table 1

We finally searched for translational evidence in support of the above findings. We used an unpublished dataset on concomitant dw-MRI and neuropsychological evaluation in AUD patients (see Methods). In good agreement with previous findings [9], FA was significantly lower in several regions of interest (ROIs) at 1–2 weeks of abstinence in patients compared to healthy subjects (Fig. 7A; the column and body of the fornix, cingulum, genu and body of corpus callosum, sagittal stratum, uncinate fasciculus, posterior thalamic radiation, corticospinal tract, the corona radiata, cerebral peduncle, internal and external capsules, superior longitudinal fasciculus, tapetum, and stria terminalis), controlling for age differences at p < 0.05 level and corrected for multiple comparisons. Interestingly, analysis of effect sizes across multiple white matter tracts showed that the fimbria/fornix has the largest reduction in FA in AUD compared to healthy controls (Figs. 7B and 51% more than the second most affected tract, the genu of the corpus callosum, and 74% more than the average of all affected tracts). Similar results were obtained applying cerebrospinal fluid (CSF) correction (Suppl. Figure 2). Because age differed between groups, in addition to controlling for age, we repeated the analysis in a subpopulation of age matched subjects, finding comparable results (HC n = 35, AUD n = 35, Mann-Whitney test: U = 488,5, p value = 0,146; Suppl. Figure 3). Thus, these results uncovered a high vulnerability of the fimbria/fornix to the deleterious effects of chronic alcohol consumption.

Finally, we computed the correlation between FA in the fimbria/fornix and the results obtained in 5 cognitive tests applied to the AUD patients in the cohort (see Methods and Suppl. Table 2). The analysis revealed significant associations with both parts of the TMT, and the NST (Suppl. Figure 4), while no significant correlations were found with the WCST and the Stroop test. Specifically, the processing time in both TMT versions was longer and the number of completed items in the NST was lower for AUD patients with a larger reduction in FA in the fimbria/fornix. Non-parametric correlation between FA and the first principal component (PC1) of the correlated cognitive variables, explaining 87% of the total variance, and partialized to account for the significant association with age of both cognitive and microstructural variables (Suppl. Figure 4, see Methods), revealed a significant negative correlation between PC1 and FA (Kendall’s Tau B = 0,216; p-value = 0,034). This result suggests that a history of severe AUD independently of age affects cognitive performance and fimbria/fornix microstructure in a correlated manner.

## Discussion

In this work, we provide causal evidence in a rat model of AUD that chronic intermittent alcohol affects the microstructural integrity of the fimbria/fornix with a functional consequence on the HC-PFC effective communication. This result gains special significance considering the high vulnerability that we find for this white matter tract in AUD patients vs. healthy controls, as measured by the decrease in FA, and its correlation with the impairment in executive function supported by the PFC. Based on our modelling work, we hypothesize that the disturbed effective connectivity from the HC to PFC in the abstinence phase would affect the learning of new contextual cues with adaptive value, impairing the extinction of previously associated alcohol memories, decreasing behavioral flexibility, and narrowing the repertoire of possible behavioral responses during abstinence.

### Myelin content in the fimbria/fornix of PD rats is decreased in alcohol abstinence

Using multimodal MRI in rats, we report a decrease in the fractional anisotropy of the fimbria/fornix, a parameter sensitive to microstructural integrity, and a decrease in the myelin water fraction, an indirect but specific [59] estimation of the myelin content in the tissue. Both results pointed to an impairment of fiber integrity after chronic exposure to alcohol in PD rats, an observation that was also reported by an independent study [60]. Furthermore, we found comparable results after one month of voluntary alcohol drinking in msP rats [9], a genetic AUD model displaying distinct phenotypic characteristics, including anxiety and propensity to negative affect [61]. Therefore, the new results extend the finding of alcohol-driven white matter alterations to a wild-type rat strain with intermittent alcohol exposure. Furthermore, they demonstrate that the fimbria/fornix alcohol vulnerability generalizes across animal models.

Conventional diffusion MRI, however, lacks specificity to different tissue sub-compartments, preventing direct interpretations on the underlying neurobiological substrates being affected [62–64]. For instance, the alterations in FA and MD found in AUD patients and rat models could reflect axonal damage, decreased myelin content or even an inflammatory glial reaction, as shown by mathematical modelling [9]. Therefore, we set out to confirm the hypothesis histologically, and found a decrease in the staining intensity of MBP. This white matter protein is required for myelin assembly [65], and its loss triggers myelin breakdown in demyelinating diseases [66, 67]. Evidence of MBP reduction was found in a study with AUD patients by western-blot analysis [68]. However, the presence of comorbid factors such as co-abuse with tobacco or liver cirrhosis, can preclude causal associations of MBP in AUD studies [69]. A recent preclinical study in mice with alcohol binge drinking also showed reduced MBP staining, in this case in the HC and PFC [70]. Our own work extends this result to the protracted abstinence stage in alcohol-dependent rats and highlights the vulnerability of the fimbria/fornix and the persistence of the myelin damage. Overall, animal studies demonstrate that bouts of binge drinking during adolescence [70] and relatively short periods (4 weeks) of excessive alcohol intake in adult rats [9], are sufficient to induce important white matter alterations which can persist long into abstinence especially in rats with a history of alcohol dependence [60].

Further research will be necessary to unveil the mechanism of demyelination. In addition to direct toxic effects of alcohol on myelinating cells [52], there is emerging evidence supporting an alcohol induced chronic inflammatory response [25, 71, 72] that affects both white and grey matter as demonstrated by an associated microglia reaction [25, 63, 73]. Also, the concept of myelin plasticity, which argues that white matter is changing permanently depending on neuronal activity was recently introduced [74]. In monkeys, decreased neuronal activity due to hippocampal lesions was associated with specific microstructural alterations in the fornix and the ventro-medial PFC white matter [75]. However, in our rat model HC activity increased during abstinence (Fig. 4), arguing against a contribution of neuronal activity to the observed microstructural changes. Regardless of the mechanism, our results unveil a causal relation between chronic effects of alcohol in rats and fimbria/fornix demyelination.

### Electrophysiological changes in the hippocampus during alcohol abstinence

We have investigated the functional consequences of the fimbria/fornix alteration using electrophysiological recordings in vivo. We found an increased excitability in alcohol abstinent vs. control conditions, both in CA1 and the DG, which corresponded, however, with a decreased efficiency in the functional coupling from the HC to the PFC. A number of electrophysiological studies have investigated, using the hippocampus as a model circuit, the effects of alcohol exposure on neurotransmission and synaptic plasticity [76–80]. Although the literature is highly variable and sometimes contradictory, it is important to note that much of this heterogeneity is likely due to distinct experimental conditions, in particular to the regimes of alcohol exposure. Overall, however, the evidence converges on at least two observations, first, at the circuit level there is a shift in the cellular excitation/inhibition balance towards more excitable states [76, 81] and, second, an initial impairment in synaptic plasticity resolves soon after alcohol withdrawal [82]. Our findings in PD rats fit well with these observations. Seven weeks of CIE followed by about two weeks of abstinence rendered granular and specially CA1 pyramidal neurons more excitable, with no detectable changes in inhibitory circuits. However, moderate effects on the baseline inhibitory tone cannot be discarded with the pair-pulse stimulation protocols used. Long-term synaptic potentiation in alcohol abstinent vs. naïve animals was comparable, as previously shown in CIE models after 5 days of abstinence [83]. Overall, the change in excitation/inhibition balance in the hippocampus together with a demyelinated fimbria/fornix, pointed to an altered hippocampus-dependent and systems-level communication.

### Effective connectivity in the fimbria fornix

The fimbria/fornix is the pathway in which hippocampal axons travel in their way to the prefrontal cortex, connecting with the prelimbic and infralimbic regions and cingulate cortex, as well as the orbitofrontal and insular cortices [84–86]. In our electrophysiological study we measured the effective connectivity from the hippocampus to the prefrontal cortex as the amplitude of the evoked activity recorded in the later in response to neuronal firing in the former, which corresponds to the polysynaptic activation from the dorsal to the ventral hippocampus and then, through the fimbria/fornix tract, to the prefrontal cortex. This preparation allowed us to measure input and output activities, as well as the directionality, providing a measure of how efficiently hippocampal activity reaches the prefrontal cortex. It is important to differentiate from functional connectivity measures, as commonly applied in fMRI or EEG studies [87], in which connectivity means correlation or coherence between two signals, with no directionality nor, indeed, demonstration of direct connection between the structures. In fact, measures of functional connectivity in those terms in the present study, like coherence and correlation analysis, found no significant difference in the HC-PFC coupling (Fig. 7D-E). The latter result should be taken with caution, as the dynamics of spontaneous activity might have been affected by anesthesia. In conclusion, lower effective connectivity in PD animals during abstinence, measured by polysynaptic activity propagation, must be interpreted as a decreased capacity of the hippocampus to condition information processing in the prefrontal cortex.

The HC projection to the PFC is important for contextual memory formation and reward learning, including cue-reward associations [88, 89] but also for extinction learning, a mechanism by which maladaptive associations can be suppressed [90]. Previous work showed that the anticipatory firing in the PFC in response to reward expectation [91–93] was indeed commanded by the hippocampal connection and prevented by its inactivation [88]. Similarly, extinguishing a fear-context association when the fear stimulus had disappeared, was dependent on the HC projection [17, 21, 94, 95] and prevented by long-term synaptic depression in the HC [94]. Also, the acquisition of a new learning rule in rats has been shown to occur concomitant with higher functional coupling between both structures (*88*), an effect that was also observed at the choice point of a spatial decision-making task in correct trials [89, 96, 97]. In agreement with these experimental results, we found, using a simple computational network model mimicking hippocampal and neocortical dynamics during memory formation, that a compromised HC-PFC communication decreases the extinction and enhances the cue-reactivation of old memories (encoded before the HC-PFC disconnection) in detriment of the recall of newly encoded memory associations. The impairment of memory updating would unavoidably decouple behavioral outputs from the changes in environmental contingencies.

Synaptic plasticity in the HC has been shown to regulate long-range connectivity in a network of mesolimbic and prefrontocortical structures including the HC, PFC and the nucleus accumbens [98–101], crucial structures in the development of addiction in general, and alcohol dependence in particular [102, 103]. Supporting a role of the HC-PFC interaction in the post-dependent phenotype, we and others found decreased performance in attentional set-shifting, a task evaluating deficits in executive functions [24, 60, 104]. PD rats showed behavioral inflexibility in this task especially at higher cognitive load. This deficit persisted for at least one month of abstinence and contributed to the increased relapse propensity in this AUD model. Overall, the microstructural alteration reported in the fimbria/fornix and impacting on the HC-PFC interaction could be central to the cognitive impairment found in alcohol dependent subjects.

### Translational evidence in AUD patients

In humans, impairment of the HC-PFC connection has been related with disorders like schizophrenia, major depression or post-traumatic stress disorder, being considered a weak link for psychiatric disorders [16]. The HC input to the PFC has been shown to be also critically important in stress regulation [105–109], a function that could link the microstructural alteration in the fornix with relapse proneness [110]. In agreement with this interpretation, we previously showed hypersensitivity of PD rats to stress and stress-triggered alcohol consumption [35]. Furthermore, the attenuated activation of dorsolateral PFC in AUD patients, which is associated with reduced cognitive flexibility produced by the delay in the extinction of non-adaptive behaviors when reinforcement contingencies change [111, 112], could be explained by a defective HC input as proposed here. The significant correlations between FA reduction in the fimbria/fornix and the reduced cognitive flexibility especially under time pressure found in AUD patients in our study, further supports this view.

White matter alterations in AUD patients have been well documented [11, 13, 113–117], they correlate with drinking levels before detoxification and progress after alcohol withdrawal during at least 6 weeks of abstinence [9]. While these alterations are widespread in the brain anatomy, they have been more consistently reported in some tracts like the corpus callosum, the fimbria/fornix, internal and external capsules and cingulate and longitudinal fasciculi [9, 11–14]. Reductions in brain volume in AUD patients, mainly associated to white matter loss [118], also show regional heterogeneity, with frontal, mesolimbic and cerebellar structures more vulnerable. We now show by formal analysis of effect sizes in a cohort of AUD patients that the fimbria/fornix presents the largest reduction in FA at 2 weeks of abstinence. This reduction was nearly double than the average of the affected tracts. Importantly, a recent study using multimodal brain imaging in a large general population (36,678 generally healthy middle-aged and older adults from the UK Biobank) [119], found consistent associations between daily alcohol units consumed and lower FA values in thirteen WM tract regions, with the strongest effects in the fornix. This observation supports the vulnerability of the fimbria/fornix to alcohol consumption.

Previous literature suggests that alterations in the white matter microstructure may explain some aspects of the cognitive decline observed in AUD patients [120–123]. For example, alterations in the corpus callosum (also reported in this work), responsible for prefrontal cortices’ connectivity [124, 125], were associated with an impairment both in executive function [120] and decision making [121]. Our results in patients provide further evidence, demonstrating a significant correlation between fimbria/fornix microstructure and the performance in cognitive tasks that explicitly rely on processing speed (NST, TMT-A, TMT-B). related to hippocampal-prefrontal function [126–131]. These results give evidence that a reduction of FA is related to cognitive performance in AUD patients in general but also particularly with executive functions like inhibitory control, a cognitive function particularly impaired in AUD patients [132] and related to increased craving and relapse risk in addicted individuals [24, 133].

Overall, the HC may have a more relevant role in the addiction cycle in AUD than commonly attributed, being central to the pathophysiology. At initial stages of alcohol exposure, an intact fimbria/fornix supports HC connections important for cue-reward association contributing to incentive learning and the stabilization of alcohol related behaviors (e.g., habituation). Later, in the absence of reward, fimbria/fornix dysfunction would interfere with the effective connectivity from the HC to the PFC, impairing learning of the new contingencies that could direct adaptive behavioral control towards resource-saving computational processes, thereby favoring habitual responding to alcohol-related stimuli.

### Limitations

One limitation of the dw-MRI technique is the contamination of white matter measurements by the proximity of the ventricles and the imaging resolution limit creating partial volume effects. The fimbria/fornix is in close contact with the third ventricle and therefore the free-water diffusion properties of the cerebrospinal fluid may have contaminated FA measurements. To mitigate this problem, we have followed the recommendations of Benear et al. 2019 [134], specifically: we have used a RESTORE approach to exclude corrupted volumes (thus mitigating the effect of brain pulsatile motion), and we have used the TBSS approach for the statistics, in order to select only the inner part of the tract. The same authors also recommend applying a correction for free water to the DTI signal before calculating the tensor to eliminate the potential contribution of the CSF [38]; while this correction is controversial when applied to the whole brain, as in ROIs far from the ventricles the free water tensor will likely pick up the signal from other isotropic cellular compartments like e.g. inflammation-associated oedema [135], in the supplementary material we show that when this correction is applied, the fimbria/fornix is still the most vulnerable tract (reduction in FA 60% larger than the average of the affected tracts) and its alteration correlates with cognitive performance in the patients.

It is also important to note that an alteration in the fimbria/fornix could also affect communication between other structures not studied here. With respect to the HC, for example, its connections with the septum and nucleus accumbens could also be compromised, which would add to the decreased effective connectivity with PFC further compromising HC function in abstinence.

Finally, the present study was performed with a relatively limited sample size and only in male subjects. However, important sex differences to alcohol exposure have been found [136, 137] that warrant further work comparing fimbria/fornix vulnerability in males and females.

## Conclusions

In summary, our results unveil a fimbria/fornix microstructural vulnerability to alcohol drinking that compromises HC-PFC communication and could explain important cognitive alterations found in the alcohol post-dependent abstinent states in humans and animal models. The HC-PFC connection would participate in the addiction cycle at two distinct moments: first, supporting reinforced learning at the onset of consumption; and later, when the fimbria/fornix is damaged, preventing the extinction of maladaptive alcohol-associated memories necessary to exit the pernicious cycle of consumption, abstinence and relapse. The latter being further facilitated by limited stress-coping abilities. Future studies will investigate the mechanisms underlying the high fimbria/fornix vulnerability to alcohol-induced white matter microstructural alterations. Clinical translation directed to repair and/or strengthen this fiber tract, maybe considering plasticity-inducing activation protocols with deep transcranial magnetic stimulation (TMS), could offer a much-needed novel therapeutic possibility in AUD.

## Electronic supplementary material

**Supplementary Figures: Supplementary Figure 1**. Short and long-term synaptic plasticity are preserved in the hippocampus of PD rats. (A) Pair pulse stimulation of the perforant pathway. Suprathreshold and equal intensity pairs of pulses (upper panel) are delivered at varying inter-stimulus time intervals (ISI). The results (lower panel) are expressed as the PS amplitude ratio (PS2/PS1). PS ratios below 1 indicate response depression and vice versa. Insets show pair pulse inhibition (left) and facilitation (right). (B) LTP of the perforant pathway. The upper panel shows the stimulation protocol used to induce LTP. Lower panel: synaptic potentiation in PD (orange) and control (black) rats quantified as the percentage increase of the PS amplitude after vs. before LTP induction. (C) Same as B but for the EPSP slope. (D) Evolution of the PS amplitude in response to perforant path stimulation before and 1 h after LTP induction. LTP protocol is applied at minute 6 (lasting 16 min) and PS amplitude is not tested until 1h later (grey shadow). The PS is normalized to pre-LTP measurements, and averaged across animals (n=5 per group). Data represents mean ± SEM. **Supplementary Figure 2**. Effect size of FA alterations in AUD patients analyzed with CSF correction (see Methods). **Supplementary Figure 3**. Effect size of FA alterations in a group of AUD patients chosen to avoid the age differences between groups. **Supplementary Figure 4**. Linear correlations between FA in the fimbria/fornix and cognitive variables: (A) NST, (B) TMT-A, (C) TMT-B, (D) number of perseverative errors in the WCST, (E) reaction times in the WCST, and (F) the Stroop test. (G) Correlation between FA in the fimbria/fornix and age. **Supplementary Table 1**. List of regions employed for the effect size analysis. **Supplementary Table 2**. List of cognitive test scores and age from AUD patients.

## Data Availability

All data needed to evaluate the conclusions in the paper are present in the paper and/or the Supplementary Materials. Additional data are available from authors upon request.

## List of abbreviations

AUD: Alcohol use disorder
dw-MRI: Diffusion-weighted magnetic resonance imaging
MRI: Magnetic resonance imaging
HC: Hippocampus
PFC: Prefrontal cortex
msP: Marchigian sardinian rats
CIE: Chronic intermittent exposure
PD: Post-dependent
NST: Number symbol test
TMT-A: Trail making test A
TMT-B: Trail making test B
BAC: Blood alcohol concentration
TR: Repetition time
TE: Echo time
MSME: Multi-slice multi-echo
FA: Fractional anisotropy
TBSS: Tract-based spatial statistics
ROI: Region of interest
MF: Myelin Fraction
AP: Anteroposterior
ML: Mediolateral
DV: Dorsoventral
DG: Dentate gyrus
LTP: Long term potentiation
PS: Population spike
EPSP: Evoked postsynaptic potential
PBS: Phosphate-buffered saline
PFA: Paraformaldehyde
CTF: Corrected total fluorescence
MBP: Myelin basic protein
LFP: Local field potential
PCA: Principal component analysis
