# Supplementary material for: Alcohol-induced damage to the fimbria/fornix reduces hippocampal-prefrontal cortex connection during early abstinence
Source: Acta Neuropathol Commun. 2023 Jun 21;11:101. doi: 10.1186/s40478-023-01597-8 (PMC10286362; doi:10.1186/s40478-023-01597-8)
Supplement: Supplementary file 1 — Supplementary Figures: Supplementary Figure 1. Short and long-term synaptic plasticity are preserved in the hippocampus of PD rats. (A) Pair pulse stimulation of the perforant pathway. Suprathreshold and equal intensity pairs of pulses (upper panel) are delivered at varying inter-stimulus time intervals (ISI). The results (lower panel) are expressed as the PS amplitude ratio (PS2/PS1). PS ratios below 1 indicate response depression and vice versa. Insets show pair pulse inhibition (left) and facilitation (right). (B) LTP of the perforant pathway. The upper panel shows the stimulation protocol used to induce LTP. Lower panel: synaptic potentiation in PD (orange) and control (black) rats quantified as the percentage increase of the PS amplitude after vs. before LTP induction. (C) Same as B but for the EPSP slope. (D) Evolution of the PS amplitude in response to perforant path stimulation before and 1 h after LTP induction. LTP protocol is applied at minute 6 (lasting 16 min) and PS amplitude is not tested until 1h later (grey shadow). The PS is normalized to pre-LTP measurements, and averaged across animals (n=5 per group). Data represents mean ± SEM. Supplementary Figure 2. Effect size of FA alterations in AUD patients analyzed with CSF correction (see Methods). Supplementary Figure 3. Effect size of FA alterations in a group of AUD patients chosen to avoid the age differences between groups. Supplementary Figure 4. Linear correlations between FA in the fimbria/fornix and cognitive variables: (A) NST, (B) TMT-A, (C) TMT-B, (D) number of perseverative errors in the WCST, (E) reaction times in the WCST, and (F) the Stroop test. (G) Correlation between FA in the fimbria/fornix and age. Supplementary Table 1. List of regions employed for the effect size analysis. Supplementary Table 2. List of cognitive test scores and age from AUD patients. [file 40478_2023_1597_MOESM1_ESM.docx]

**Supplementary Figures**


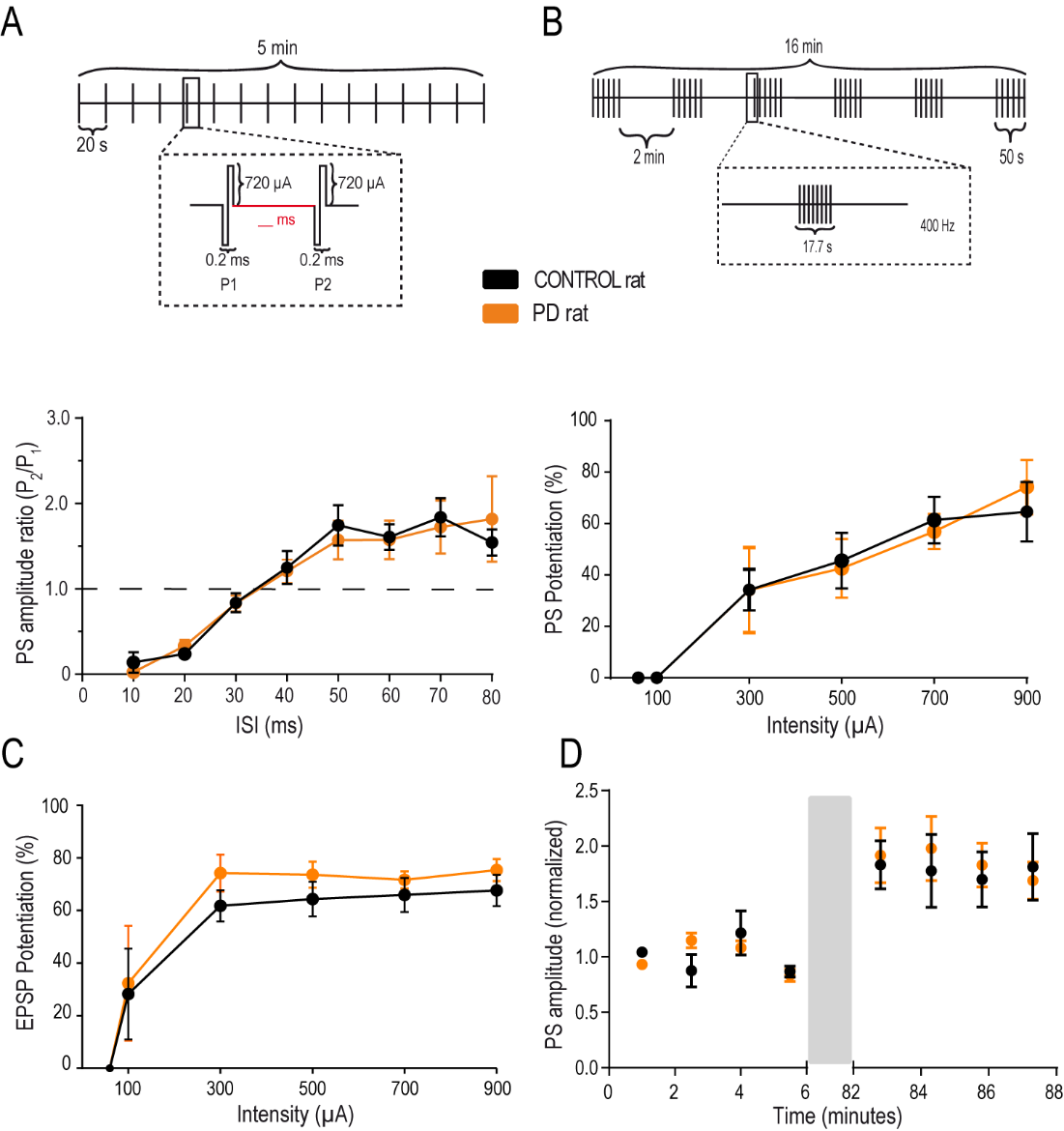


**Supplementary Figure 1.** Short and long-term synaptic plasticity are preserved in the hippocampus of PD rats. **(A)** Pair pulse stimulation of the perforant pathway. Suprathreshold and equal intensity pairs of pulses (upper panel) are delivered at varying inter-stimulus time intervals (ISI). The results (lower panel) are expressed as the PS amplitude ratio (PS2/PS1). PS ratios below 1 indicate response depression and *vice versa*. Insets show pair pulse inhibition (left) and facilitation (right). **(B)** LTP of the perforant pathway. The upper panel shows the stimulation protocol used to induce LTP. Lower panel: synaptic potentiation in PD (orange) and control (black) rats quantified as the percentage increase of the PS amplitude after *vs*. before LTP induction. **(C)** Same as B but for the EPSP slope. **(D)** Evolution of the PS amplitude in response to perforant path stimulation before and 1 h after LTP induction. LTP protocol is applied at minute 6 (lasting 16 min) and PS amplitude is not tested until 1h later (grey shadow). The PS is normalized to pre-LTP measurements, and averaged across animals (n=5 per group). Data represents mean ± SEM.


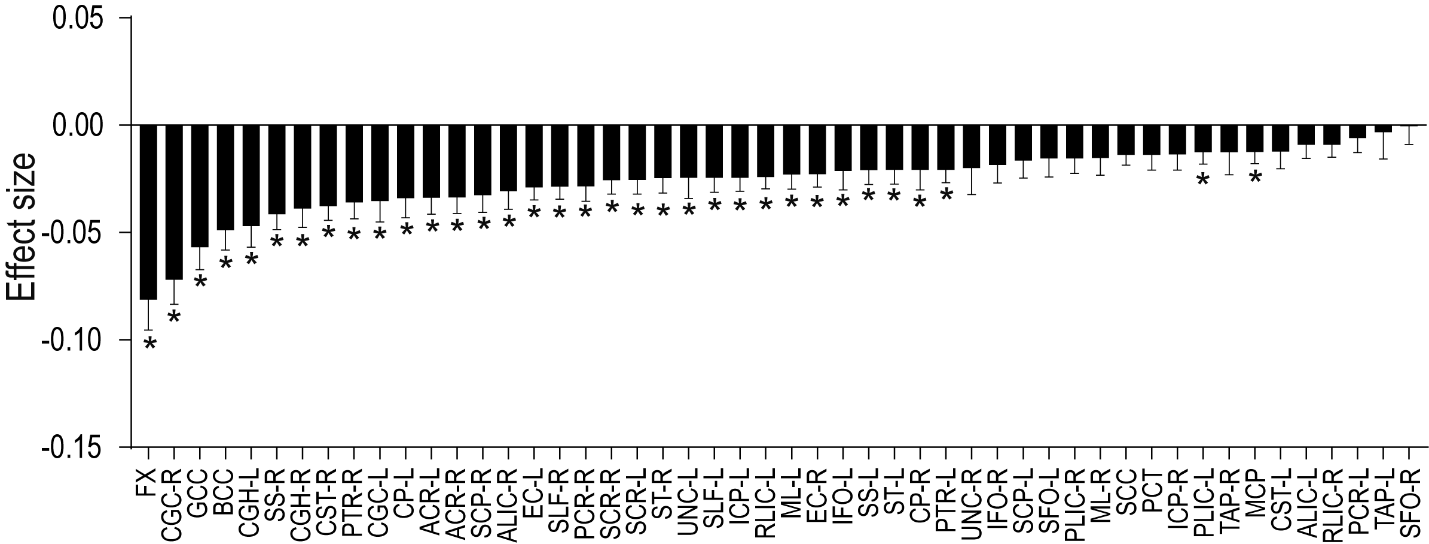


**Supplementary Figure 2.** Effect size of FA alterations in AUD patients analyzed with CSF correction (see Methods).


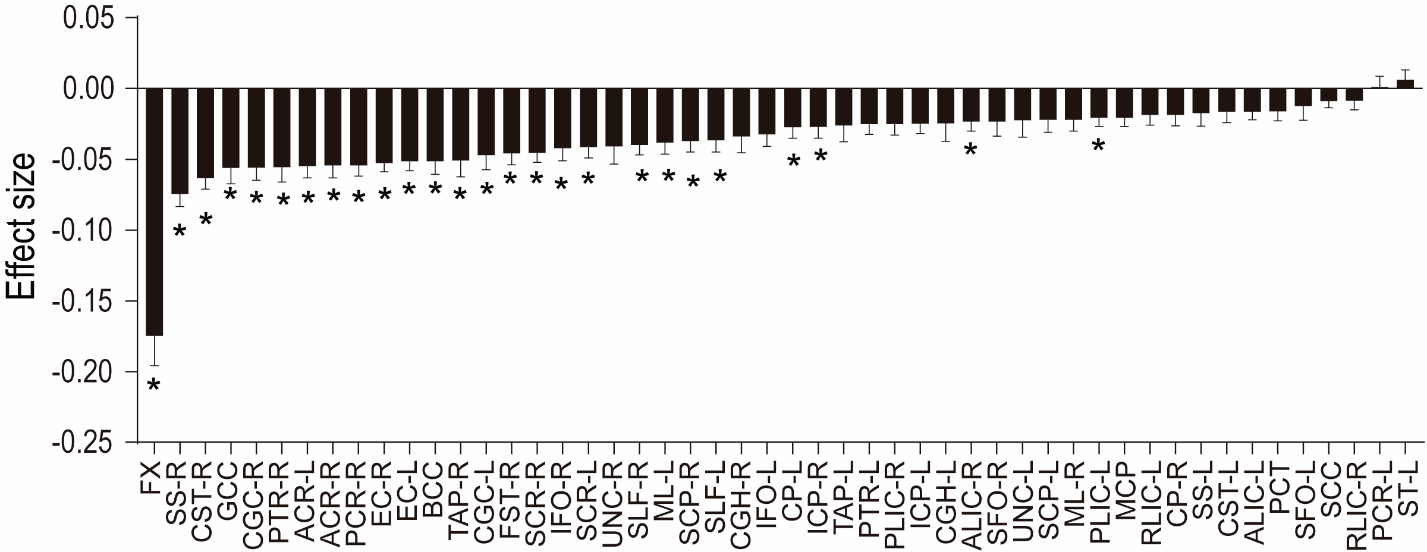


**Supplementary Figure 3.** Effect size of FA alterations in a group of AUD patients chosen to avoid the age differences between groups.


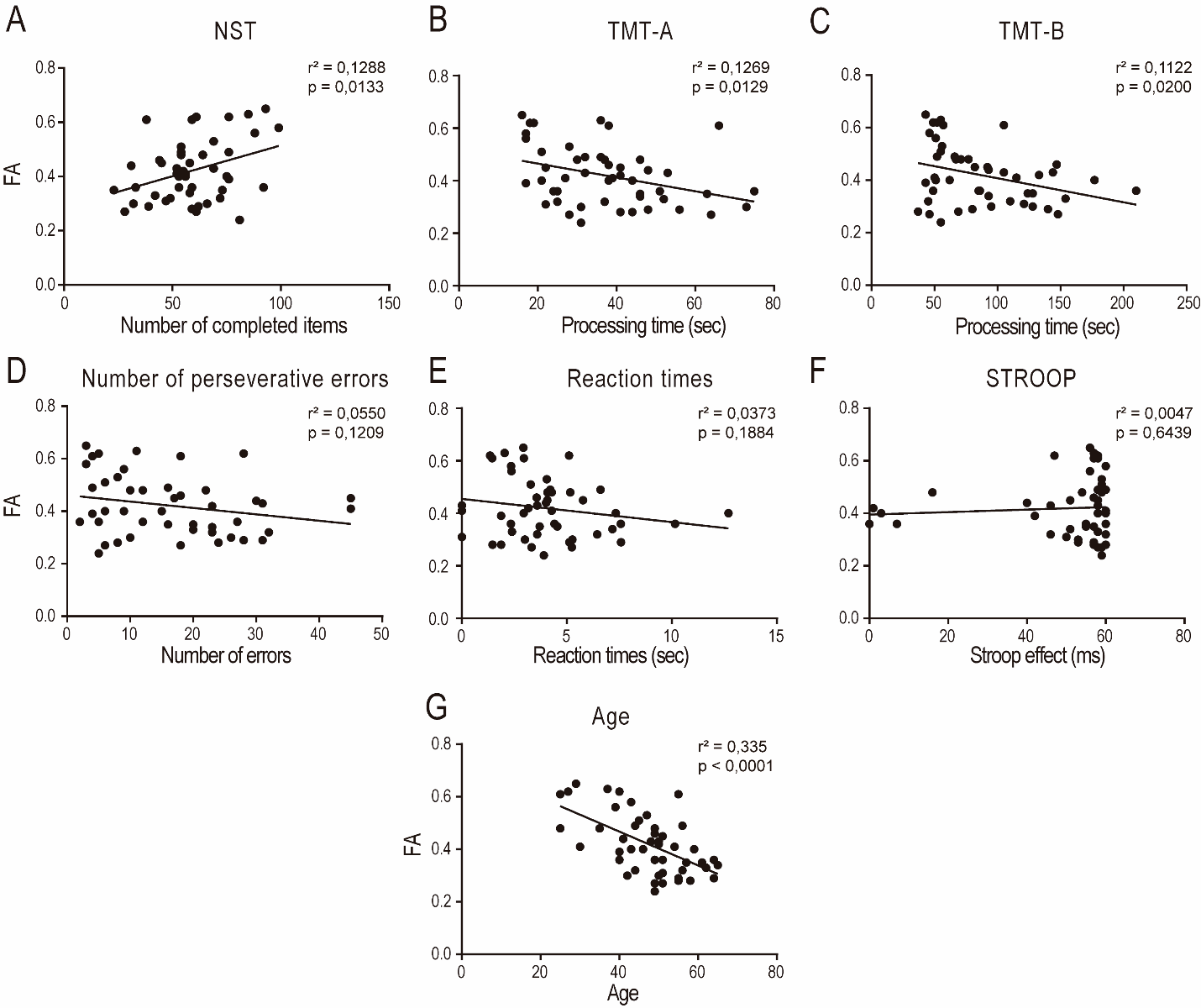


**Supplementary Figure 4.** Linear correlations between FA in the fimbria/fornix and cognitive variables: **(A)** NST, **(B)** TMT-A, **(C)** TMT-B, **(D)** number of perseverative errors in the WCST, **(E)** reaction times in the WCST, and **(F)** the Stroop test. **(G)** Correlation between FA in the fimbria/fornix and age.

**Supplementary Table 1**. List of regions employed for the effect size analysis.

| Abbreviation | Name of the brain region |
| --- | --- |
| MCP | Middle cerebellar peduncle |
| PCT | Pontine crossing tract (a part of MCP) |
| GCC | Genu of corpus callosum |
| BCC | Body of corpus callosum |
| SCC | Splenium of corpus callosum |
| FX | Fornix (column and body of fornix) |
| CST-R | Corticospinal tract right |
| CST-L | Corticospinal tract left |
| ML-R | Medial lemniscus right |
| ML-L | Medial lemniscus left |
| ICP-R | Inferior cerebellar peduncle right |
| ICP-L | Inferior cerebellar peduncle left |
| SCP-R | Superior cerebellar peduncle right |
| SCP-L | Superior cerebellar peduncle left |
| CP-R | Cerebral peduncle right |
| CP-L | Cerebral peduncle left |
| ALIC-R | Anterior limb of internal capsule right |
| ALIC-L | Anterior limb of internal capsule left |
| PLIC-R | Posterior limb of internal capsule right |
| PLIC-L | Posterior limb of internal capsule left |
| RLIC-R | Retrolenticular part of internal capsule right |
| RLIC-L | Retrolenticular part of internal capsule left |
| ACR-R | Anterior corona radiata right |
| ACR-L | Anterior corona radiata left |
| SCR-R | Superior corona radiata right |
| SCR-L | Superior corona radiata left |
| PCR-R | Posterior corona radiata right |
| PCR-L | Posterior corona radiata left |
| PTR-R | Posterior thalamic radiation (include optic radiation) right |
| PTR-L | Posterior thalamic radiation (include optic radiation) left |
| SS-R | Sagittal stratum (include inferior longitudinal fasciculus and inferior fronto-occipital fasciculus) right |
| SS-L | Sagittal stratum (include inferior longitudinal fasciculus and inferior fronto-occipital fasciculus) left |
| EC-R | External capsule right |
| EC-L | External capsule left |
| CGC-R | Cingulum (cingulate gyrus) right |
| CGC-L | Cingulum (cingulate gyrus) left |
| CGH-R | Cingulum (hippocampus) right |
| CGH-L | Cingulum (hippocampus) left |
| ST-R | Stria terminalis right |
| ST-L | Stria terminalis left |
| SLF-R | Superior longitudinal fasciculus right |
| SLF-L | Superior longitudinal fasciculus left |
| SFO-R | Superior fronto-occipital fasciculus (could be a part of anterior internal capsule) right |
| SFO-L | Superior fronto-occipital fasciculus (could be a part of anterior internal capsule) left |
| IFO-R | Inferior fronto-occipital fasciculus right |
| IFO-L | Inferior fronto-occipital fasciculus left |
| UNC-R | Uncinate fasciculus right |
| UNC-L | Uncinate fasciculus left |
| TAP-R | Tapetum right |
| TAP-L | Tapetum left |

**Supplementary Table 2**. List of cognitive test scores and age from AUD patients.

| **Age** | **NST Completed items** | **TMT-A**  **(sec)** | **TMT-B**  **(sec)** | **WCST Number of perseverative errors** | **WCST Reaction times (sec)** | **Stroop effect (ms)** |
| --- | --- | --- | --- | --- | --- | --- |
| 25 | 38 | 66 | 105 | 18 | 2,95 | 58 |
| 35 | 54 | 37 | 71 | 10 | 4,08 | 54 |
| 40 | 76 | 17 | 43 | 4 | 1,871 | 42 |
| 29 | 93 | 16 | 43 | 3 | 2,932 | 56 |
| 51 | 47 | 22 | 121 |  | 0 | 50 |
| 37 | 85 | 36 | 55 | 11 | 2,037 | 57 |
| 47 | 69 | 28 | 56 | 8 | 4,045 | 59 |
| 55 | 59 | 38 | 57 | 4 | 1,442 | 57 |
| 55 | 39 | 48 | 140 | 28 | 5,12 | 53 |
| 55 |  | 44 | 37 | 24 | 1,87 | 57 |
| 59 | 53 | 38 | 177 | 15 | 7,326 | 58 |
| 58 | 59 | 41 | 69 | 8 | 1,463 | 60 |
| 45 | 54 | 21 | 55 | 6 | 3,276 | 59 |
| 49 | 33 | 75 | 210 | 12 | 7,571 | 7 |
| 64 | 53 | 25 | 85 | 5 | 10,15 | 55 |
| 51 | 58 | 41 | 92 | 45 | 4,07 | 58 |
| 39 | 88 | 17 | 51 | 9 | 2,361 | 56 |
| 43 | 99 | 17 | 46 | 3 | 2,349 | 60 |
| 65 | 58 | 46 | 93 | 23 | 7,171 | 51 |
| 50 | 69 | 53 | 144 |  | 0 | 46 |
| 51 | 45 | 22 | 82 | 17 | 5,774 | 51 |
| 49 | 81 | 31 | 55 | 5 | 3,903 | 59 |
| 49 | 61 | 28 | 46 | 6 | 3,321 | 58 |
| 30 | 52 | 27 | 50 |  | 0 | 60 |
| 40 | 92 | 24 | 49 | 2 | 2,336 | 60 |
| 40 | 61 | 19 | 52 | 5 | 1,344 | 58 |
| 42 | 66 | 31 | 128 | 10 | 3,002 | 53 |
| 56 | 54 | 36 | 66 | 16 | 6,602 | 58 |
| 51 | 59 | 51 | 86 | 27 | 4,412 | 0 |
| 51 | 28 | 64 | 148 | 18 | 5,237 | 59 |
| 25 | 64 | 46 | 77 | 12 | 4,276 | 59 |
| 49 | 64 | 30 | 67 | 22 | 5,166 | 16 |
| 48 | 52 | 32 | 105 | 31 | 3,59 | 58 |
| 57 | 73 | 46 | 128 | 20 | 3,704 | 55 |
| 50 | 32 | 73 | 95 | 26 | 5,266 | 53 |
| 44 | 49 | 37 | 110 | 32 | 6,442 | 46 |
| 56 | 72 | 25 | 45 | 23 | 3,59 | 60 |
| 44 | 76 | 32 | 52 | 4 | 4,211 | 60 |
| 49 | 44 | 38 | 147 | 18 | 3,564 | 57 |
| 43 | 75 | 21 | 51 | 9 | 12,693 | 60 |
| 62 | 42 | 52 | 154 | 20 | 2,384 | 58 |
| 50 | 55 | 41 | 133 | 23 | 3,17 | 1 |
| 61 | 23 | 63 | 124 | 16 | 4,549 | 57 |
| 46 | 56 | 44 | 62 | 6 | 2,946 | 3 |
| 64 | 62 | 56 | 80 | 31 | 7,577 | 57 |
| 41 | 31 | 48 | 93 | 30 | 4,011 | 40 |
| 27 | 76 | 18 | 49 | 28 | 5,104 | 47 |
| 54 | 56 | 39 | 115 | 45 | 4,283 |  |
